# Microfluidic Single-Cell Bioanalysis for Decoding Tumor Heterogeneity

**DOI:** 10.3390/bios16090473

**Published:** 2026-08-28

**Authors:** Xingyu Tao, Shuang Feng, Yi Luo, Zhenfei Yu, Ruiheng Wang, Ru-Jia Yu

**Affiliations:** 1 State Key Laboratory of Flexible Electronics (LoFE), Institute of Advanced Materials (IAM), Nanjing University of Posts and Telecommunications, Nanjing 210023, China; b23070310@njupt.edu.cn (X.T.);; 2 Department of Hematology and Hematopoietic Cell Transplantation, City of Hope National Medical Center, Los Angeles, CA 91010, USA

**Keywords:** microfluidic bioanalysis, single-cell analysis, tumor heterogeneity, source attribution, extracellular vesicles, clinical validation

## Abstract

Tumor heterogeneity drives cancer progression, dissemination, therapeutic adaptation, and relapse, yet many clinically relevant cell states are rare, transient, context-dependent, and obscured by population-averaged analysis. This review examines how microfluidic platforms preserve biologically meaningful linkages among cell identity, molecular state, secreted output, functional phenotype, perturbation history, and microenvironmental context, which are frequently disrupted by conventional workflows. We first define analytical requirements imposed by tumor heterogeneity, then examine microwell- and microchamber-based systems, droplet microfluidic platforms, valve-assisted and other active manipulation or capture systems, and integrated multimodal workflows that preserve single-cell information while introducing distinct engineering trade-offs. We further discuss major readout modalities, including genomic and transcriptomic profiling, extracellular vesicle and secretome analysis, metabolic measurements, and proteomic readouts, and applications in circulating tumor-cell dissemination, tumor–microenvironment interactions, and therapy-response heterogeneity. Finally, we highlight bottlenecks in measurement fidelity, source attribution, reproducibility, benchmarking, biological representation, multimodal integration, and clinical validation. We propose that microfluidic single-cell oncology should advance from descriptive profiling toward decision-oriented systems that preserve cell-resolved states, source-attributed outputs, perturbation histories, and longitudinal responses within reproducible workflows and connect them to clinically actionable information.

## 1. Introduction

**Figure 1:**
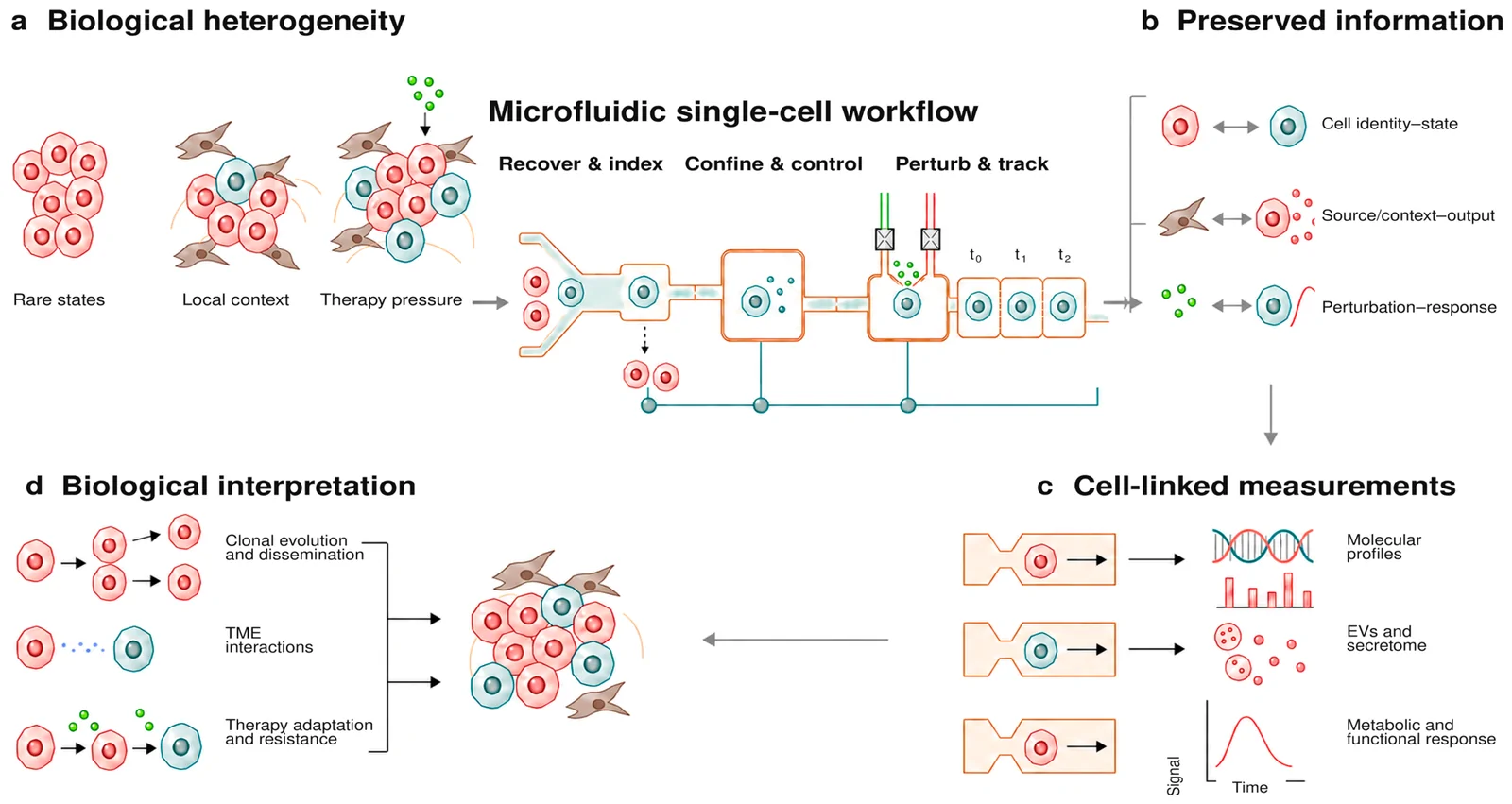
Conceptual framework showing how microfluidic single-cell workflows preserve biological linkage from tumor heterogeneity to cell-linked measurements and biological interpretation.

Tumor heterogeneity creates a fundamental analytical challenge in cancer research and precision therapy [1]. Clinically important tumor-cell states are often rare, transient, spatially localized, and shaped by microenvironmental and therapeutic conditions [2]. These states may include invasive subpopulations, therapy-tolerant persister cells, immune-evasive phenotypes, circulating tumor cells, and cells with altered secretory or metabolic programs [3]. Although population-averaged assays remain useful for defining dominant molecular features, they compress heterogeneous cell populations into composite signals and often fail to reveal which cells carry the most consequential traits [4]. Single-cell approaches are therefore often essential, but tumor heterogeneity also imposes technical demands that different platforms meet with varying success [5,6]. Isolating individual cells is only part of the challenge. Rare populations must be recovered with minimal loss, sample processing should minimize handling-induced state distortion, locally secreted or short-lived signals must remain attributable to their cellular source, and experimental perturbation should remain coupled to downstream molecular or functional readouts [7]. Microfluidic systems address these requirements through controlled confinement, fluid handling, temporal regulation, and multistep workflow integration, enabling weak, local, or transient signals to be captured before they are diluted or lost (Figure 1) [8].

Device categories alone do not reveal whether a workflow preserves cell identity, signal source, perturbation history, and downstream response [9]. Existing reviews have summarized microfluidic technologies for single-cell analysis from perspectives such as device fabrication, droplet- and microwell-based compartmentalization, cell sorting strategies, on-chip detection modalities, and high-throughput molecular assays, while others have emphasized cancer-oriented applications including circulating tumor-cell enrichment, tumor-on-chip and organoid systems, extracellular vesicle analysis, and microfluidic drug screening [10,11]. Although these studies provide valuable overviews of technological development and application landscapes, they remain largely organized around platform architecture, assay type, or sample category [12,13,14]. Existing classifications leave unresolved whether cellular origin, secretion, perturbation history, and downstream response remain linked throughout the workflow, even though this linkage determines biological attribution and clinical interpretation [15,16].

To address this gap, this review follows a structured progression, from tumor heterogeneity as a biological and analytical challenge to microfluidic strategies for preserving single-cell information, to multimodal readouts capturing complementary layers of tumor biology, and finally to biomedical applications and translational constraints. Within this framework, we integrate tumor biology, microfluidic engineering, and single-cell analytical modalities into a unified perspective centered on biological information preservation. In this review, “single-cell analysis” encompasses not only direct measurements of isolated individual cells, but also microfluidic workflows in which cellular origin, clonal derivation, or cell-resolved heterogeneity remains experimentally traceable. Low-input or multicellular models are included only when they preserve or interrogate heterogeneity at a cell-resolved or clonally attributable level. Unlike reviews organized primarily by device architecture, assay format, or application domain, we evaluate microfluidic strategies according to the biological relationships they retain, the biases they introduce, and the consequences for interpretation and translation.

## 2. Tumor Heterogeneity as an Analytical Challenge for Microfluidic Single-Cell Analysis

**Figure 2:**
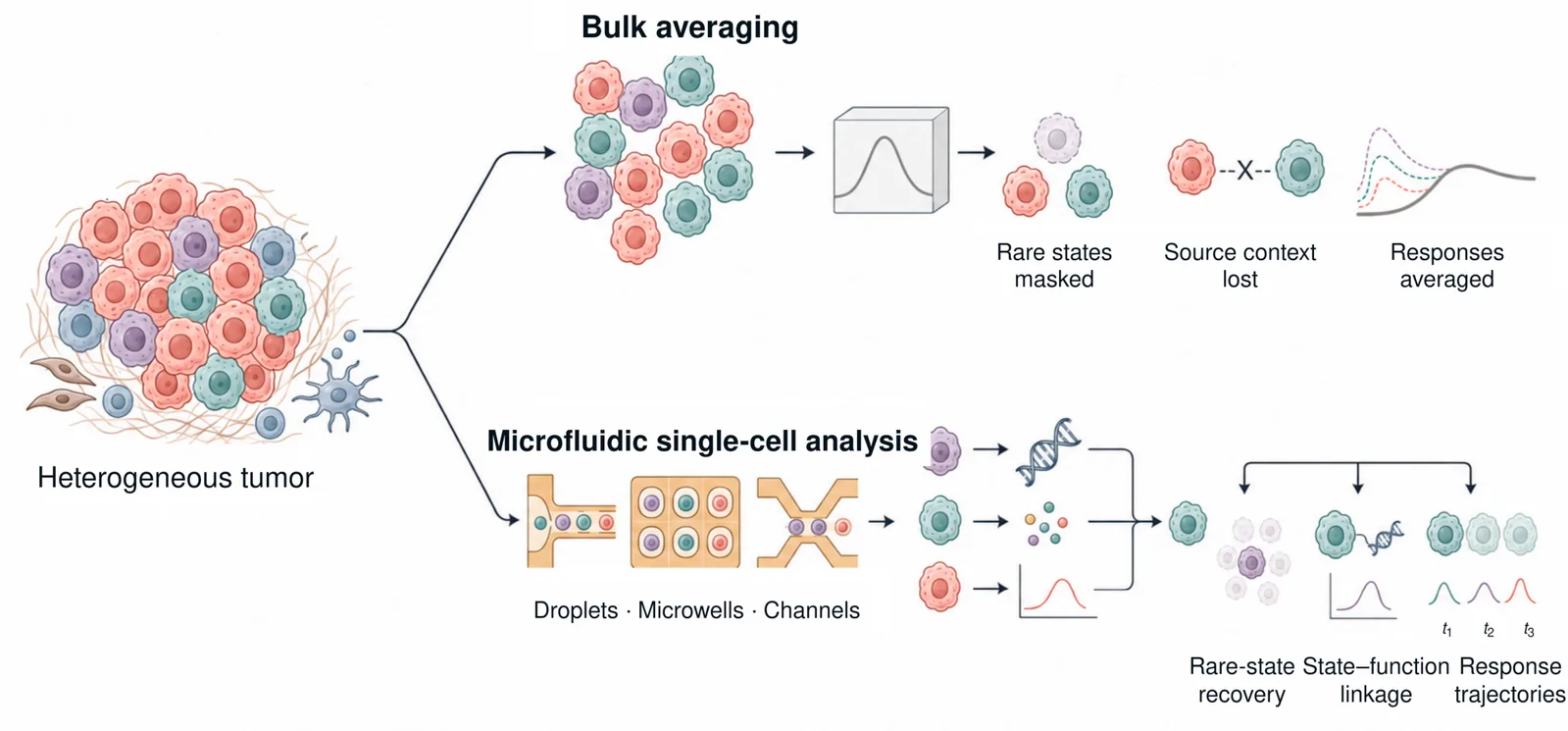
Microfluidic single-cell analysis preserves rare cellular states, cell-linked measurements, and individual response trajectories that are obscured by bulk averaging.

Tumor heterogeneity is not confined to a single layer of tumor biology. What varies within a tumor includes lineage structure, genetic and epigenetic state, transcriptional program, cellular behavior, secretory activity, metabolic preference, and local ecological context (Figure 2). These layers do not contribute equally to every biological or clinical question, but together they shape how tumors grow, disseminate, evade immune pressure, adapt to therapy, and relapse. For microfluidic analysis, the key challenge is to retain the relationships among cell identity, cellular state, functional behavior, local context, and treatment response before they are obscured during sample processing and measurement [17,18,19].

### 2.1. Biologically Consequential Dimensions of Tumor Heterogeneity

One major dimension of tumor heterogeneity is variation in clonal architecture and cellular state. Tumor cells differ in mutations, copy-number alterations, and broader chromosomal abnormalities, and these features define the clonal structure through which tumor evolution proceeds [20,21]. However, clonal structure alone does not capture the full extent of intratumoral diversity. Genetically related cells can diverge in chromatin accessibility, DNA methylation, transcriptional programs, and stress response states, which means that cells within the same clone may differ substantially in phenotype and behavior [22,23,24]. These state differences become particularly consequential when they translate into functional divergence. Individual tumor cells can vary in proliferative capacity, migratory and invasive behavior, secretory activity, metabolic preference, and stress tolerance [25]. Such functional differences often have more immediate consequences for tumor progression than molecular differences alone would suggest. For example, a rare subpopulation with increased invasive capacity or enhanced stress tolerance may have limited influence on the average molecular profile of a tumor, but it may disproportionately contribute to dissemination, relapse, or treatment failure. Tumor heterogeneity therefore cannot be understood solely by cataloging molecular states. It also requires methods that can connect these states to measurable cellular behavior under defined conditions [26].

The functional consequences of heterogeneity are further shaped by spatial and ecological context. Oxygen tension, nutrient availability, extracellular matrix composition, stromal interaction, and immune infiltration are unevenly distributed across tumor tissue and continuously influence cellular behavior. Heterogeneity emerges from the interaction between cell intrinsic programs and local microenvironmental conditions, and this interaction becomes particularly important under therapeutic pressure [27]. Treatment rarely produces a uniform response. Some cells are eliminated, some enter transient adaptive states, and others persist in forms that later support relapse [3]. Therapy therefore exposes and reshapes pre-existing differences in clonal structure, regulatory plasticity, functional capacity, and ecological support, bringing their consequences for treatment response into sharper view [28].

### 2.2. Limits of Population-Averaged Analyses

These dimensions of tumor heterogeneity highlight the limitations of population-averaged analyses. Resolution is only one constraint. More fundamentally, bulk measurements collapse signals from clonally, transcriptionally, and functionally distinct cells into a single composite output [29,30]. As a result, they obscure the cellular origin of measured signals, dilute rare but consequential cell states, and flatten differences that may be more biologically important than the average readout [31]. This matters especially in tumors, where many clinically relevant behaviors arise from minority populations. Cells with increased invasiveness, altered secretion, enhanced stress tolerance, immune evasive capacity, or early treatment resistance may represent only a small fraction of the tumor-cell population, yet they can disproportionately shape progression and relapse. Bulk assays can still reveal dominant trends, but they are poorly suited to identifying which subpopulations carry these traits or how such traits are distributed across the tumor [26]. The implication for single-cell analysis is clear. The relevant question is not simply how many distinct cell states can be detected, but which relationships among genotype, state, behavior, microenvironment, and treatment response remain experimentally traceable. Platform performance is better judged by whether genotype, state, behavior, microenvironment, and treatment response remain traceable to the same cell or sample.

The limitation becomes even more pronounced when the relevant biology involves state transitions over time. Population-averaged measurements do not by themselves distinguish whether a measured signal reflects a stable dominant state, a mixture of divergent states, selective expansion of a minority population, or a transient adaptive response emerging only in a subset of cells. Once averaging compresses this temporal divergence into a single value, it becomes difficult to reconstruct the trajectories of cellular change. Bulk assays remain useful for broad profiling and for identifying dominant molecular trends, but they are poorly suited to assigning mechanisms, trajectories, or functional consequences to specific cell populations. This does not mean that bulk analysis should be replaced in all settings. Rather, it indicates where single-cell and microfluidic approaches add distinct value. They are most needed when the biological question requires source attribution, rare population recovery, longitudinal tracking, controlled perturbation, or linkage between molecular state and functional behavior. In this sense, the limitation of population-averaged analysis is not only the loss of resolution, but also the loss of relationships among cells, signals, behaviors, and context.

Tumors exhibit heterogeneity across multiple dimensions, including clonal and cell-state diversity, functional and ecological variation, and therapy-driven evolution. Bulk analysis averages these differences across mixed populations and therefore masks rare states, obscures source-resolved information, and flattens dynamic divergence. Microfluidic single-cell analysis addresses these limitations by preserving cell identity, cellular state, source-attributed outputs, perturbation history, and longitudinal response within controlled microscale workflows.

### 2.3. Microfluidic Requirements for Preserving Single-Cell Tumor Information

**Figure 3:**
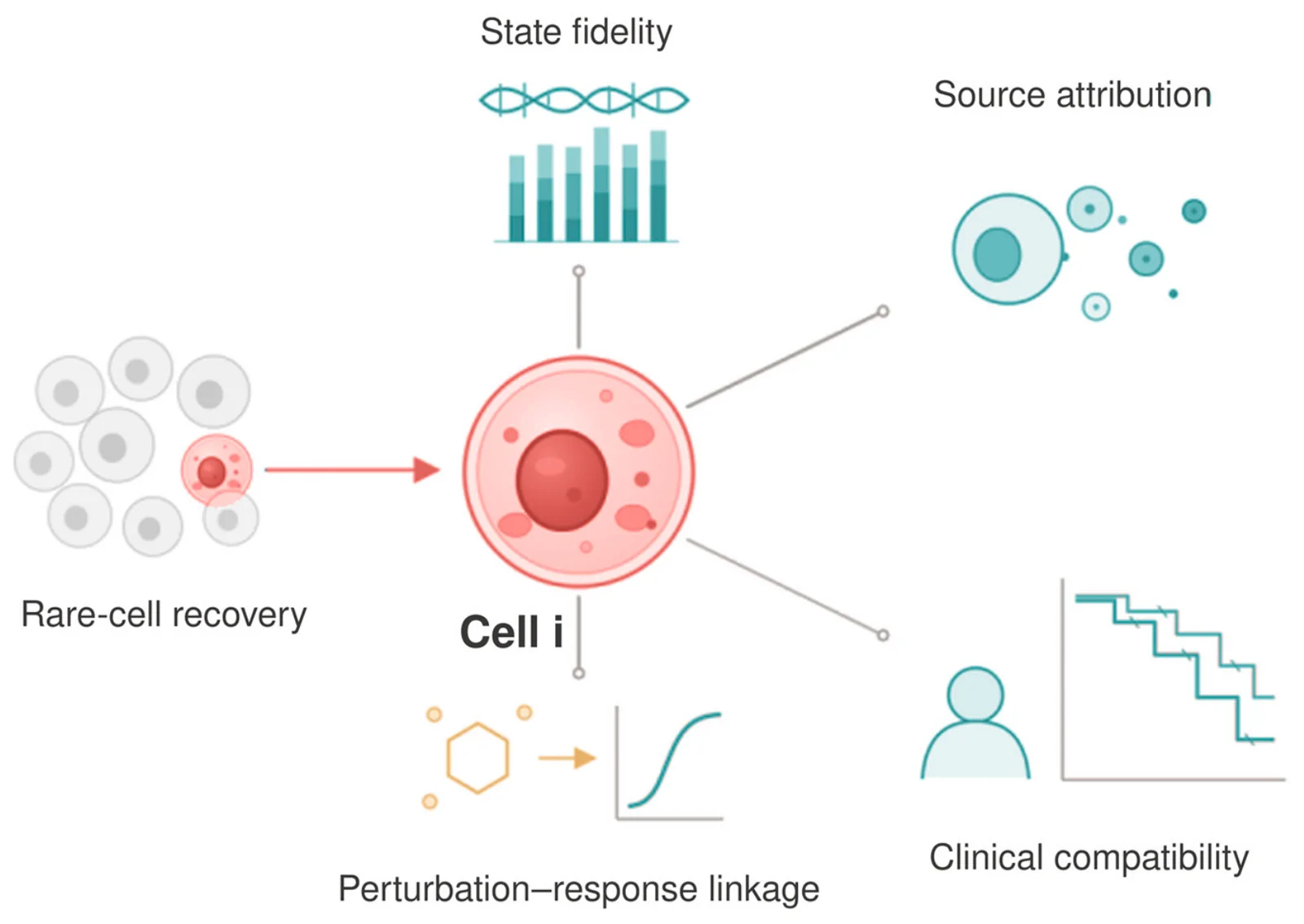
Key requirements for interpretable microfluidic single-cell analysis: rare-cell recovery, state fidelity, source attribution, perturbation–response linkage, and clinical compatibility. Here, “Cell i” denotes a representative indexed single cell whose identity is preserved throughout the analytical workflow.

Tumor heterogeneity cannot be interpreted from single-cell measurements alone. Biological meaning depends on retaining the cellular and experimental context from which each signal arose [28]. Microfluidic workflows may preserve these connections, but they may also interrupt them. Figure 3 identifies where information can be lost between sample entry and the conclusions drawn from the resulting data. Rare populations can be depleted during enrichment, and the cells that remain may be altered during isolation or confinement [32,33]. This problem is particularly relevant to CTC enrichment, where the physical properties selected by a device determine which cells enter the dataset [34]. Recovery is therefore a sampling decision as much as a technical performance metric. Viability and handling effects raise a different concern: even when a cell is retained, it may no longer represent its state in the original specimen. The dataset can consequently acquire a biological bias before downstream measurement begins.

Further losses can arise after cells have entered the device. Molecular measurements may remain detectable after their cellular or clonal origin has been obscured, while secreted products can mix before they are assigned to a source. Phenotypic behavior becomes difficult to interpret when the preceding cellular state or local environment is missing. Drug responses pose a related problem because dose, timing, and exposure history may be separated from the measured outcome. Figure 3 marks five recurrent breaks in this chain. They overlap in practice rather than dividing tumor biology into independent categories. Confinement-induced stress, for instance, can alter molecular state, function, and treatment response simultaneously. A cell removed from its spatial niche may change its behavior without producing an obvious technical failure. Microfluidic control over space, time, and signal localization can limit some of these disruptions [35], but the retained signal still derives its meaning from tissue and microenvironmental context [36].

Preserving a connection does not establish that the resulting interpretation is correct. Before biological conclusions are considered, the workflow has to be examined for incomplete recovery, calibration error, leakage, cross-contamination, and poor reproducibility. A technically stable result may still reflect stress or selection introduced by the device. Orthogonal measurements can help distinguish such artifacts from biological variation. Clinical claims require outcome-linked evidence, whether the proposed endpoint is treatment response, dissemination, resistance, or recurrence. The right-hand gate in Figure 3 separates these different evidentiary demands. Computational correction may reduce batch effects, but it cannot reconstruct a cell identity, spatial position, or exposure history that was never retained experimentally.

Throughput alone is an incomplete basis for comparing microfluidic platforms. Processing more cells can improve statistical coverage while sacrificing temporal history, spatial context, or downstream recovery. Platforms designed for greater experimental control may preserve these features but operate at a smaller scale and with greater instrumental complexity. Device geometry and material interfaces shape much of this trade-off. Flow determines the population presented for analysis, whereas adsorption and mass transport influence whether cellular products remain measurable. Matrix properties may change the phenotype itself, and the detection limit determines which events enter the final dataset. Table 1 is not intended as a comprehensive catalogue of microfluidic platforms. Instead, it provides a design-oriented overview of selected engineering elements that directly influence whether single-cell information is retained, distorted or lost during the analytical workflow. These elements encompass substrate and surface properties, flow and separation architectures, spatial confinement and active fluidic control, recognition and matrix interfaces, droplet compartmentalization, and sensing interfaces. The categories are not mutually exclusive, and an individual platform may integrate several of these elements. Recent representative studies have therefore been added to illustrate emerging implementations, including semipermeable capsules for multistep single-cell processing, droplet-based combinatorial indexing for multimodal sequencing, aptamer-assisted functional screening, and hydrogel-encapsulated patient-derived tumor models. Platform-level device architectures and their suitability for different biological questions are discussed separately in Section 3.1 and Table 2.

## 3. Microfluidic Design Principles for Preserving Single-Cell Tumor Information

Microfluidic single-cell analysis centers on the ability to isolate, confine, perturb, and interrogate individual cells under controlled conditions. In tumor heterogeneity research, these engineering choices are not neutral technical details. They determine which forms of single-cell information remain preserved, which relationships are lost, and which biological interpretations become possible. Microwell and microchamber systems preserve spatial continuity, droplet platforms preserve compartmental attribution, valve-assisted systems preserve procedural continuity, optical and electrical interfaces convert preserved cellular states into measurable signals, and integrated workflows preserve linkage across analytical steps [55,56]. Each format preserves a different form of information continuity and introduces distinct failure modes. Their limitations are equally important, because each strategy introduces its own artifacts, including diffusion loss, compartment-induced stress, limited reagent exchange, reduced phenotypic breadth, calibration burden, and difficulty in downstream recovery. These limitations are biologically consequential because they can determine which cells and signals remain represented in the final dataset. Loss of rare cells, distortion of transient states, or disruption of cell–environment relationships can therefore change the apparent structure of tumor heterogeneity rather than simply reduce analytical performance.

### 3.1. Strategies for Single-Cell Isolation, Confinement, and Manipulation

**Figure 4:**
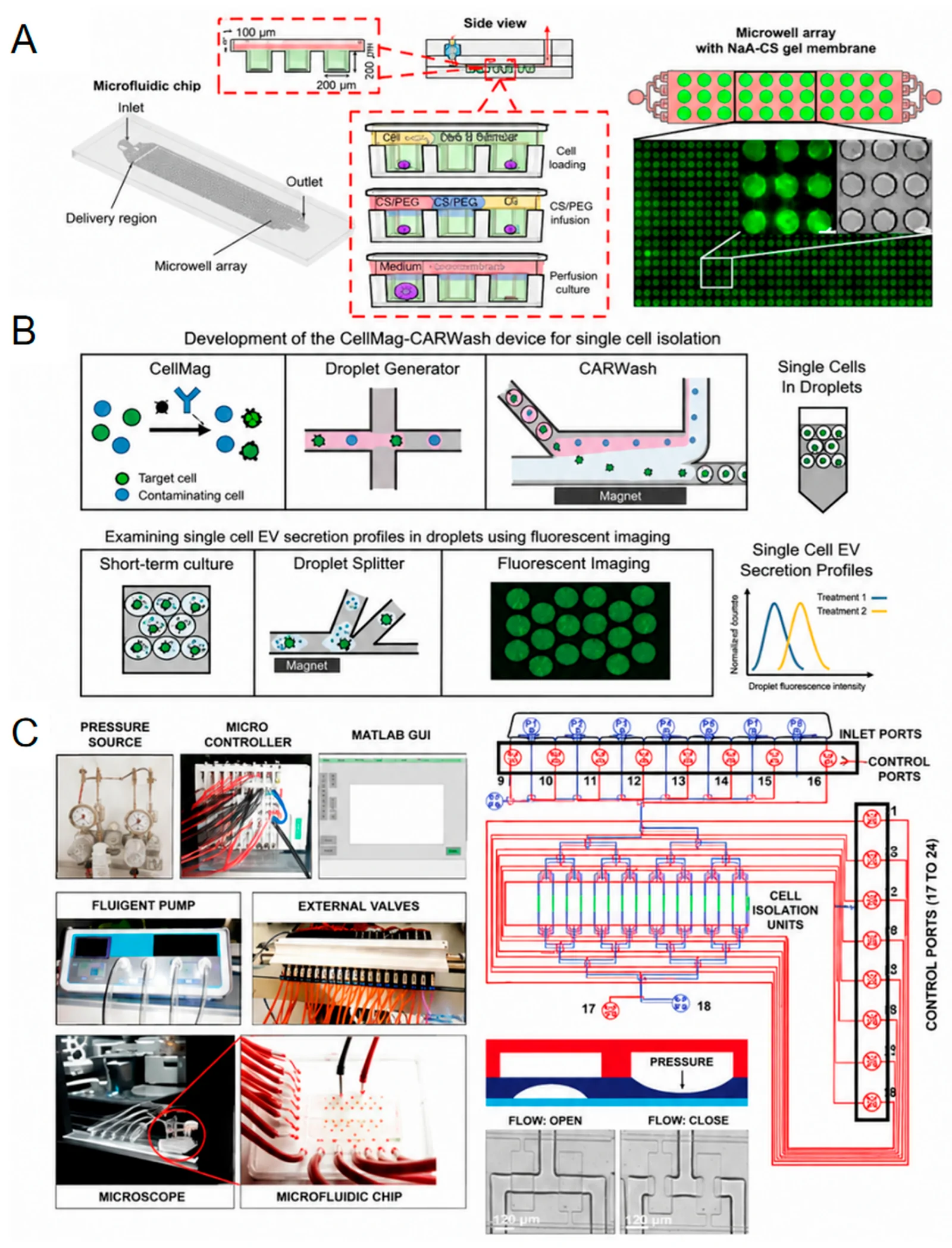
Representative microfluidic strategies for preserving heterogeneity-related single-cell information. (**A**) Microwell- and microchamber-based systems preserve spatial identity and support clonal culture, localized treatment, and image-compatible longitudinal analysis within defined compartments. Reproduced in part from Ref. [32]. Copyright 2025, the author(s). (**B**) Droplet microfluidic platforms support single-cell purification, encapsulation, compartmentalized culture, and downstream functional analysis, including fluorescence-based assessment of extracellular vesicle (EV) secretion profiles. Reproduced in part from Ref. [51]. Copyright 2024, the authors. Published by Wiley-VCH GmbH. Licensed under CC BY 4.0. (**C**) Valve-assisted active microfluidic systems provide programmable control over flow routing, cell loading, isolation, and multistep handling through integrated pneumatic actuation and switchable open/closed flow states. Reproduced in part from Ref. [63]. Copyright 2022, the author(s). Licensed under CC BY 4.0.

The first engineering decision in single-cell analysis concerns how individual cells are isolated, confined, and manipulated. In microfluidic systems, confinement directly shapes what can later be measured and how confidently outputs can be attributed to a given cell. It affects whether cellular origin remains traceable, whether diffusible products are retained near the producing cell, whether perturbations can be imposed in a defined sequence, and whether the same cells can be followed over time [57]. For this reason, strategies for isolation and manipulation are better distinguished by the type of information continuity they preserve, including spatial indexing, compartmental separation, and procedural control, rather than by device format alone [58]. Figure 4 illustrates these three forms of information continuity and shows how each becomes relevant to a different aspect of tumor heterogeneity.

#### 3.1.1. Microwell- and Microchamber-Based Approaches

Microwell- and microchamber-based formats are especially useful when cells or their clonal progeny must remain spatially indexed over time. Their core strength is positional continuity, which keeps single-cell origin linked to clonal expansion, repeated imaging, and later phenotype comparison [59]. This design is most informative when the biological question depends on spatial identity, lineage continuity, and longitudinal observation. Chen et al. developed a microfluidic chip containing 30,000 microwells for single-cell culture of colorectal cancer cells and used it to generate single-cell-derived colon cancer organoid arrays. Here, the value of the microwell format was the preservation of clonal origin during organoid formation at a scale unattainable by manual isolation [60]. Lin et al. built on this approach, moving from derivation to comparison by using a microwell-array platform to produce large-scale single-cell-derived tumor organoid arrays for colorectal cancer drug screening. Their system maintained thousands of single-cell-derived tumor organoids (STOs) at predefined positions on the same focal plane, enabling automated high-content imaging while preserving derivation from individual tumor stem cells. In this way, spatial indexing keeps clonal origin traceable while allowing phenotypic differences among clonally derived units to be compared under the same experimental conditions (Figure 4A) [32].

The main limitation is that spatial indexing does not automatically preserve all types of single-cell information. Microwell and microchamber systems are well suited to clonal tracking and repeated observation, but rapidly diffusing secreted proteins, metabolites, and EVs can be lost unless additional sealing, local capture, or volume-reduction strategies are introduced. Sequential perturbation can also be less straightforward than in valve-assisted systems. Microwells best support spatial indexing, lineage tracking, and longitudinal imaging. Studies of rapidly diffusing products or repeated perturbations require additional sealing, capture, or fluid-control features [55]. These losses can affect biological interpretation when secretory or metabolic differences define the phenotype of interest. A cell may remain spatially traceable while its rapidly released products are incompletely retained, weakening the apparent linkage between clonal identity and functional output and potentially underrepresenting short-lived secretory states.

#### 3.1.2. Droplet Microfluidics for High-Throughput Single-Cell Compartmentalization

Droplet microfluidics are built around the generation of large numbers of independent microscale compartments. Its distinctive contribution is physical compartmentalization, in which each cell remains coupled to a local and separated microenvironment. This design enables parallel processing at scale while preserving diffusible outputs close to the producing cell [50]. Droplet systems are therefore most useful when the analytical goal is to retain source attribution for secreted products, EVs, enzymatic activity, or sequencing-linked molecular profiles. Liu et al. developed an all-aqueous droplet system for single pancreatic tumor cells and used it to generate pancreatic cancer organoids at scale. The workflow produced thousands of organoids in a single batch and achieved improved uniformity and controllability compared with conventional matrix-based culture. In this context, droplet compartmentalization provided a scalable route to standardized single-cell-derived tumor units [48]. This compartmental logic is also central to secretion and EV analysis, because diffusible products lose cellular attribution once they disperse into a shared medium. Rupp et al. combined positive magnetic selection with droplet microfluidics in the CellMag-CARWash workflow for live-cell isolation and downstream single-cell analysis. Confinement within droplets enabled EV secretion from individual MCF7 cells to be measured and revealed heterogeneous changes in EV release after β-estradiol stimulation. By retaining diffusible products within cell-specific compartments, the platform preserved their association with the producing cell and made cell-to-cell differences in secretory output directly resolvable (Figure 4B) [51]. Encapsulation efficiency introduces a related constraint when the available cell number is limited. Increasing the starting cell concentration can reduce empty droplets, but it can also compromise cell focusing and increase multiple-cell encapsulation. Tang et al. addressed this trade-off by placing a tunable on-chip sample-enrichment module between spiral focusing and droplet generation, allowing cells to be focused at a lower concentration and concentrated immediately before encapsulation. The system achieved a single-cell encapsulation rate of 72.2% for MDA-MB-231 cells, showing that sample conditioning upstream of droplet formation can improve usable single-cell occupancy without relying simply on a higher input concentration [61]. More recent designs have begun to integrate upstream cell selection directly with downstream droplet compartmentalization. Galogahi et al. developed a microfluidic platform that combines inertial size-based cell separation with single-cell encapsulation in droplets within the same device. This integration reduces the discontinuity between heterogeneous-sample processing and compartment generation, illustrating a shift from droplet formation as an isolated operation toward workflows in which cell selection and encapsulation are coordinated before single-cell analysis [62].

The main trade-off is that droplet independence does not automatically support experimental continuity. Prolonged imaging, repeated reagent exchange, recovery of the same viable cell, and multistep manipulation are usually more difficult in droplet systems than in indexed or valve-assisted formats. Droplet platforms also require careful control of encapsulation statistics, doublet rate, droplet leakage, surfactant biocompatibility, and downstream recovery. Their biological value depends on whether compartment integrity, cell viability, and downstream linkage remain sufficient over the required assay period. In practice, droplet formats are generally better suited to short-term compartmentalized measurements than to prolonged longitudinal observation unless reagent exchange and viable-cell recovery are specifically engineered. These effects can directly alter the heterogeneity inferred from droplet-based measurements. Doublets can merge signals from different cells, whereas leakage weakens source attribution across compartments; in addition, confinement or surfactant-related stress may generate cellular responses that are difficult to distinguish from genuine secretory or adaptive states. Limited recovery and follow-up also make it harder to determine whether an observed state is transient or persists after the initial measurement.

#### 3.1.3. Valve-Assisted and Other Active Manipulation Approaches

Valve-assisted and other active manipulation systems are distinguished by programmable control over the timing of reagent delivery, the sequencing of perturbations, and the execution of multiple operations on the same cell over time. Their defining contribution is procedural continuity, which allows perturbation, measurement, and follow-up operations to remain coupled to the same cell within a controlled workflow. This design is most useful when temporal control or sequential manipulation is central to the biology being measured. Active manipulation is particularly valuable when the biological question requires perturbation history, measurement timing, and downstream response to remain linked within the same single-cell workflow. Valve-assisted systems can impose defined stimulation patterns, control reagent delivery, and repeatedly monitor the same confined cells, making them suitable for dynamic response analysis rather than only endpoint measurement. Representative apoptosis-dynamics platforms illustrate this advantage by using programmable pneumatic control to trap single cells, deliver temporally defined inputs, and monitor caspase-3 activation during drug-induced apoptosis in real time. By keeping stimulation history and response linked to the same cell, such control makes differences in response timing and trajectory experimentally distinguishable rather than reducing them to a population-level endpoint (Figure 4C) [63]. In the same system, more than 75% of trapped cells remained in place for 6 h, cell viability stayed above 80% over 20 h with medium exchange, and apoptosis dynamics were followed continuously for up to 16 h. This example shows that the main value of active control is not simply automation, but the preservation of temporal linkage between stimulus and response. Programmable handling also becomes important when single-cell information must remain connected across sequential analytical operations. Integrated electrical-identification and drug-screening workflows show how cancer-cell classification and downstream treatment response can be performed on the same low-input platform without transferring scarce cells between disconnected assays [64]. Compared with purely temporally focused systems, this type of workflow highlights another advantage of active manipulation: procedural continuity, in which cell identification, perturbation, and response measurement remain chained within one platform.

**Figure 5:**
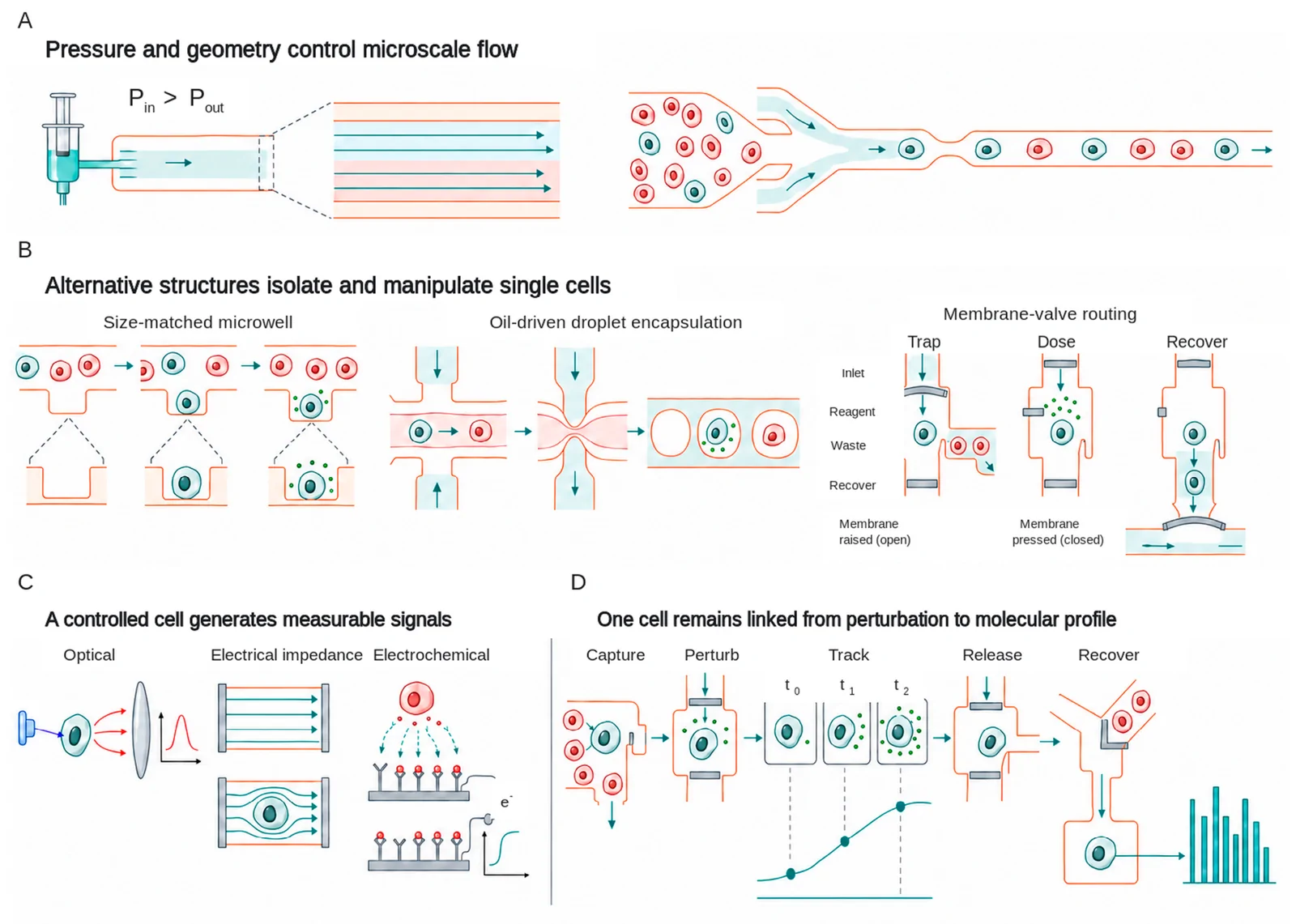
Microfluidic structure–function principles linking single-cell handling to cell-resolved measurement and downstream profiling. (**A**) Pressure gradients and channel geometry regulate microscale flow and cell positioning. (**B**) Microwells, flow-focusing droplets, and membrane-valve networks provide alternative strategies for single-cell isolation, compartmentalization, manipulation, and recovery. (**C**) Controlled single cells can be interrogated optically, electrically, or electrochemically to convert cell-associated differences into measurable signals. (**D**) Integrated workflows preserve same-cell linkage across capture, perturbation, temporal tracking, release, recovery, and downstream molecular profiling.

These gains in programmability come with practical trade-offs. Active systems are well suited to dynamic perturbation studies, repeated response measurements, and multistep workflows, but they usually require more complex fabrication, external actuation, precise control of dead volume, and lower throughput than passive compartmentalization formats. Their value is greatest when the biological question requires timed stimulation, controlled reagent exchange, or repeated measurement of the same cells. This comparison indicates that platform selection should be guided by the type of single-cell information that must be preserved. Microwell and microchamber systems are most suitable when spatial identity, clonal continuity, and longitudinal observation are central to the biological question. Droplet systems are more suitable when diffusible products, including EVs, metabolites, or secreted proteins, must remain confined near the producing cell. Valve-assisted systems are most useful when the timing of stimulation, reagent exchange, and response measurement must remain under precise experimental control. These formats should not be viewed as a hierarchy of technical sophistication. They represent different solutions to distinct information-preservation problems in tumor heterogeneity analysis. For heterogeneity studies, these constraints affect more than operational efficiency. Lower throughput can restrict the number of cells sampled and thereby reduce coverage of rare subpopulations, whereas poorly controlled dead volume or timing can blur the temporal relationship between stimulation and response. This is particularly important when transient or asynchronous treatment responses are the biological feature of interest. At the device level, these functions arise from the control of microscale flow and cell position by channel geometry, and from architectures such as microwells, droplets, and valve networks that isolate, compartmentalize, or route individual cells (Figure 5A,B).

### 3.2. On-Chip Detection and Integrated Workflows

After single cells have been isolated or confined, the next challenge is to convert preserved cellular information into reliable analytical signals. In microfluidic systems, detection is not only a downstream measurement step, but also part of the platform design. Sensor position, reaction volume, optical path, electrode geometry, capture surface, and connection to off-chip analysis all influence whether the measured signal remains attributable to the original cell or compartment. For this reason, on-chip detection strategies should be evaluated according to three criteria: whether they preserve single-cell attribution, whether they support temporal or multimodal linkage, and whether their outputs can be calibrated reproducibly across devices. Current systems mainly implement these goals through optical and fluorescence-based acquisition, electrochemical and electrical readouts, and integrated workflows that connect cell handling, perturbation, signal acquisition, and downstream analysis [65]. Figure 5 places these analytical routes in their structural context, showing how pressure and channel geometry guide cell trajectories, how microwells, droplets, and valves isolate or route individual cells, how sensing interfaces convert cell-associated events into measurable signals, and how integrated workflows can retain same-cell linkage across perturbation, tracking, recovery, and downstream profiling.

#### 3.2.1. Optical and Fluorescence-Based Signal Acquisition

**Figure 6:**
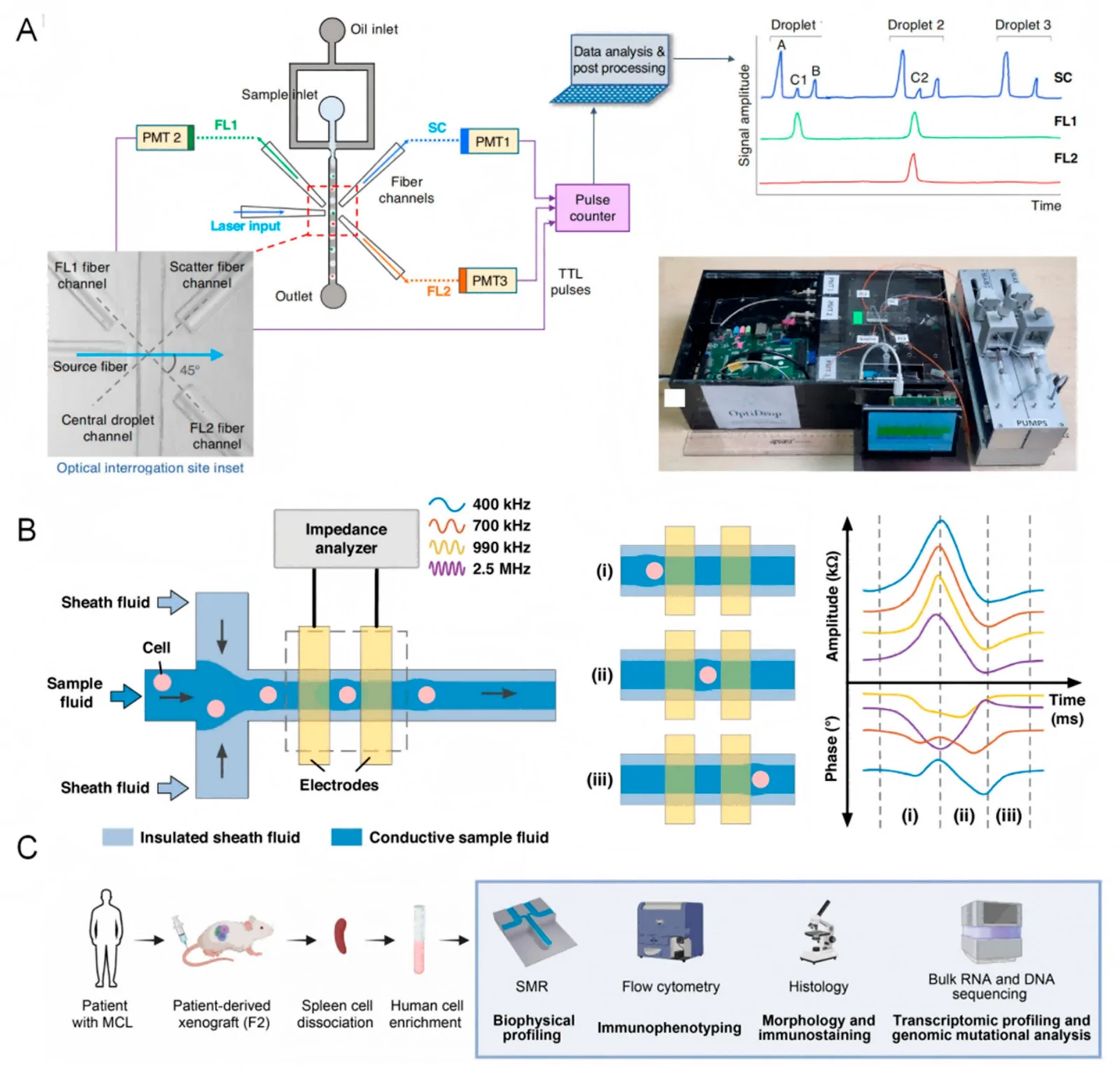
Representative on-chip detection strategies and integrated workflows in microfluidic single-cell analysis. (**A**) Optofluidic microfluidic platform integrating droplet handling with fiber-assisted optical interrogation. Laser excitation, scattered-light collection, and multichannel fluorescence detection are coupled to downstream pulse counting and post-processing, enabling real-time signal acquisition from individual droplets or droplet-encapsulated cells. Reproduced in part from Ref. [66]. Copyright 2024, the author(s). Licensed under CC BY 4.0. (**B**) Microfluidic impedance-based electrical readout for label-free single-cell interrogation. Hydrodynamic focusing guides cells through an electrode-defined sensing region, where multi-frequency impedance measurements capture amplitude and phase changes associated with cell transit and biophysical heterogeneity. Here, (**i**), (**ii**), and (**iii**) indicate the cell positions before, within, and after the impedance-sensing region, respectively. Reproduced in part from Ref. [69]. Copyright 2024, the author(s). Licensed under CC BY 4.0. (**C**) Integrated chip-enabled workflow linking patient-derived samples to multimodal downstream analysis. Following sample preparation and enrichment, microfluidic measurements are combined with complementary assays to connect biophysical profiling with molecular and functional characterization. Reproduced in part from Ref. [73]. Copyright 2025, the author(s). Licensed under CC BY 4.0.

Optical and fluorescence-based readouts offer a highly flexible approach for directly visualizing confined cells, clones, or droplets as analytical units. At the device level, this requires a defined optical interrogation region that preserves the association between the measured signal and the corresponding cell or compartment (Figure 5C). Depending on platform design, they can support immediate multiparametric acquisition, repeated observation of the same cells over time, or high-content phenotyping without staining-related perturbation. Gupta et al. demonstrated immediate multiparametric acquisition with the OptiDrop platform, which integrated optical fibers into a droplet microfluidic chip. Using a single laser and multiple photomultiplier tubes, the system enabled on-chip detection of scatter and multiplexed fluorescence signals from droplets and their contents at single-cell resolution. This enabled discrimination of droplets containing zero, one, or multiple cells and profiling of antibody-labeled surface markers on encapsulated cells [66]. Here, droplet microfluidics combined compartmentalization with integrated optical measurement (Figure 6A). Longitudinal perturbation studies require optical designs that can follow the same cells or clones over repeated measurements. Wang et al. addressed this with a high-density microchamber array coupled to an on-chip concentration-gradient generator for drug testing in single leukemia cells and single-cell-derived clones. Repeated fluorescence imaging with calcein-AM and PI staining enabled continuous observation of single cells and derived clones over a 72 h window, allowing their heterogeneous responses to different drug conditions to be compared longitudinally [67]. Optical analysis becomes more expansive when the aim shifts from labeled detection to high-content phenotyping without staining-related perturbation. Shi et al. developed the spatial microfluidic holographic integrated platform by combining spatial hydrodynamic focusing with digital holographic microscopy. The system enabled label-free quantitative phase imaging of multiple cancer cell lines and breast cancer subtypes without digital refocusing and, when combined with machine learning, achieved high classification accuracy across cancer cell types, breast cancer subtypes, and blood cells [49,68]. Here, optical analysis no longer depended on predefined fluorescent labels to resolve phenotypic variation.

Across these platforms, optical readout supports multiple forms of single-cell observability, including rapid multiparametric detection, longitudinal monitoring, and label-free phenotyping. The main limitation is that analytical richness often depends on labeling strategy, imaging conditions, phototoxicity control, and downstream computational interpretation. Restricted marker panels can leave biologically distinct states unresolved, while inadequate sampling frequency may miss short-lived transitions or make asynchronous responses appear more uniform than they are. Repeated imaging may itself perturb sensitive cells if phototoxicity is not controlled, complicating the distinction between intrinsic state changes and measurement-induced effects. Optical readouts are therefore powerful when morphological, spatial, or fluorescence-based information is central, but their biological interpretation remains sensitive to assay design and image-analysis pipelines.

#### 3.2.2. Electrochemical and Electrical Readout Strategies

Electrochemical and electrical readouts complement optical detection when the priority is label-free measurement, compact device architecture, and direct signal quantification. They convert cell-dependent electrical changes into measurable outputs without requiring optical reporters. This makes them especially useful when compact architecture, direct quantification, and integrability take precedence over high-content imaging. In tumor heterogeneity research, they provide a distinct route for capturing cell-to-cell differences through quantitative electrical signatures. Impedance-based microfluidic analysis illustrates this approach. In impedance-based systems, channel focusing and electrode geometry define the sensing region through which individual cells generate discrete electrical events (Figure 5C). Wang et al. developed a microfluidic impedance flow cytometer that used the crossflow of conductive sample fluid and insulating sheath fluid to create a virtual constriction microchannel above embedded electrodes. Within this design, individual K562, Jurkat, and HL-60 cells generated distinguishable impedance pulses, and the resulting signals supported highly accurate cell classification when coupled to a recurrent neural network [69]. In this case, electrical readout functioned as a compact label-free discriminator embedded within a microfluidic architecture (Figure 6B). Electrical readouts gain biological value when they are calibrated against behaviorally relevant phenotypes rather than used only for cell classification. Jiang et al. moved in this direction by integrating electrochemical impedance measurement with microfluidic and Transwell-based elements for the evaluation of HeLa cell invasiveness. In their system, impedance variation was used to assess invasion-associated behavior and to build a quantitative model relating signal response to invasive capacity [70]. Under this design, electrical readout became a quantitative proxy for a cancer-relevant cellular property rather than only a means of label-free classification. A more recent development has extended impedance-based single-cell analysis toward direct evaluation of clinical tumor specimens. Hui et al. developed an integrated circuit-based single-cell electric cell–substrate impedance sensing platform for rapid discrimination between cancerous and non-cancerous tumors. The system was evaluated using clinical tumor samples and illustrates the progression of electrical microfluidic analysis from cell-line classification and phenotype-associated measurements toward rapid analysis of patient-derived material [71].

Electrochemical and electrical strategies are most useful when single-cell differences must be translated into compact and directly quantifiable signals. Their limitation is that signal compactness often comes at the expense of phenotypic breadth and molecular specificity. Electrical signatures can reflect size, membrane properties, deformability, adhesion, viability, or local microenvironmental changes, and their biological interpretation depends on calibration against orthogonal molecular or functional measurements. Consequently, distinct biological states may produce overlapping electrical signatures, whereas similar classifications may arise from different underlying mechanisms. Without orthogonal validation, apparent electrical heterogeneity should therefore not be interpreted directly as molecular or functional heterogeneity. Electrical readouts favor speed, integration, and label-free operation, but their biological meaning requires calibration against molecular or functional measurements.

#### 3.2.3. Integrated Workflows for Multistep Single-Cell Analysis

Microfluidic systems are also valuable because they can reduce the discontinuities that usually separate sample handling, perturbation, measurement, and downstream analysis. In single-cell studies of tumor heterogeneity, those discontinuities matter because analytical value is often lost when input material is limited or when different types of information must remain linked to the same cells. Here, integration is valuable only if the same biological unit remains identifiable across successive operations; capture, perturbation, temporal tracking, release, and recovery must therefore remain linked if downstream molecular profiles are to be interpreted together with the preceding functional response (Figure 5D). Integrated workflows become especially valuable when genetic, secretory, metabolic, and functional differences must be resolved from the same limited sample. Chen et al. developed a rapid microfluidic drug sensitivity testing system that supported spheroid formation and drug screening from minimal primary tumor material within 5 days. The platform enabled both single-drug and combination-drug testing without prior expansion and was applied to primary samples from 21 patients with breast cancer. By integrating sample preparation, microscale 3D culture, drug perturbation, and viability-based evaluation within one workflow, the system preserved operational continuity and reduced manipulations that might otherwise reshape the sample before response was assessed [72]. This requirement becomes more demanding when continuity must be preserved across both experimental operations and information layers. Zhang et al. coupled suspended microchannel resonator measurements with downstream single-cell RNA sequencing in mantle cell lymphoma. In this workflow, buoyant mass and stiffness were measured first, and the same cells were then collected for transcriptomic profiling [73]. This allowed biophysical variation to be interpreted alongside gene expression programs at single-cell resolution in primary and patient-derived xenograft samples, linking mass and stiffness to clinically relevant phenotypes and transcriptional features associated with oncogenic B cell receptor signaling (Figure 6C). Recent integrated platforms have also expanded the number of functional dimensions that can be followed within the same single-cell workflow. Shao et al. developed the High-throughput Single-Cell Omni-functional Profiling Engine (HiSCOPE), which combines high-density single-cell trapping with modular assays for proliferation, drug susceptibility, cytokine secretion, cytotoxicity, and defined cell–cell interactions. Importantly, selected viable cells can be recovered after on-chip functional interrogation for subsequent analysis. This design extends integrated microfluidics from linking a small number of predefined measurements toward preserving multiple functional phenotypes and downstream access to the same cells [74].

Integrated workflows preserve informational continuity across steps that are often experimentally separated. Their main value is that distinct biological readouts remain attributable, comparable, and jointly interpretable. However, integration also increases system complexity. Each additional module can introduce sample loss, calibration error, time delay, or batch effect. These errors can accumulate across modules and reshape the apparent relationship between information layers. Selective cell loss may preferentially remove scarce states, while delays between measurements can decouple rapidly changing molecular or functional phenotypes from the state recorded at an earlier step. Batch- or module-specific variation can likewise create apparent multimodal differences that do not reflect biological heterogeneity. Therefore, integrated platforms should be evaluated not only by the number of readouts they combine, but also by whether linkage across readouts remains reliable at the level of the same cell, clone, compartment, or patient-derived sample.

When patient material is scarce, collecting as many readouts as possible is not necessarily the best use of the sample. The more practical approach is to start with the measurement that is most directly tied to the question and add another layer only when it helps explain the first. In drug-response studies, this may mean following the functional response over time and then profiling the same cell or indexed unit molecularly at the endpoint. For rare CTCs, establishing which cells were recovered and what state they are in is generally more informative than dividing a small sample across several downstream assays. The same logic applies to TME models: preserving the interacting cells and their behavior should come first, with secretome or molecular analysis added when the aim is to explain the interaction. EV or secretome measurements are therefore most useful when intercellular communication itself is the question. Under severe sample constraints, a focused two-layer design will often provide a clearer biological answer than a broader multimodal workflow.

Across these examples, platform architecture and interfacial design jointly determine the type of single-cell information that can be preserved. Spatially indexed formats are suited to clonal tracking and longitudinal imaging, whereas compartmentalized droplets strengthen source attribution for diffusible products. Valve-assisted systems provide temporal control over perturbation and reagent delivery. Capture interfaces enrich rare cells or EVs from limited specimens, and electrode- or transducer-integrated systems translate cellular behavior into quantitative physical or molecular signals. These formats should be viewed not as a hierarchy of device complexity, but as distinct solutions to different analytical requirements in single-cell tumor analysis. Table 2 reorganizes these strategies around the biological question to be addressed, linking the heterogeneity to be resolved with the relevant microfluidic principle, an appropriate device design or methodology, and the resulting measurement or application. The table is intended to help readers identify which microfluidic strategy is better matched to a given research question, while also indicating when the information required by that question may exceed what a single platform can preserve or measure reliably. The central question now shifts from how single cells are handled and preserved to which layer of tumor heterogeneity can be recovered, measured, and interpreted. Device architecture therefore defines the information that remains experimentally accessible, but it does not by itself determine what biological conclusion can be drawn. That depends on the analytical readout applied to the preserved cells, compartments, or signals.

**Figure 7:**
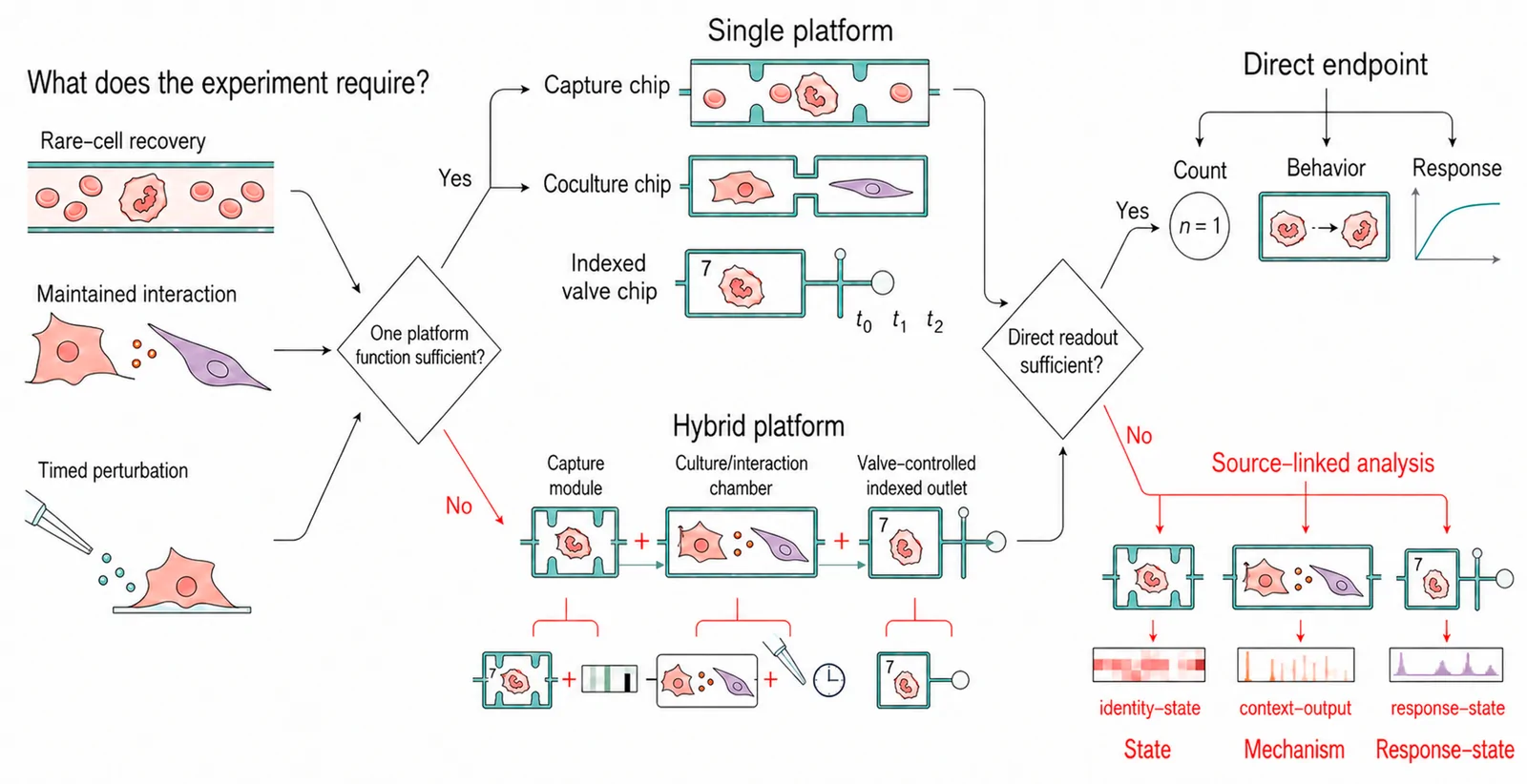
Question-guided selection of microfluidic platforms and hybrid workflows for single-cell tumor analysis. The two decision points distinguish when a single platform and direct readout are sufficient from when online functional integration or source-linked analysis is required. Red elements denote the workflow branches and source-linked outputs used when a single platform or direct readout is insufficient.

In practice, platform selection depends on which relationships must remain intact through the experiment. Rare CTC-state analysis usually begins with enrichment, but capture alone does not establish cell state; phenotypic or molecular profiling is needed when the aim is to distinguish biologically or clinically relevant circulating subpopulations. TME crosstalk places a different priority on preserving interacting cells together with their spatial or temporal context, so paired-cell, co-culture, or spatially indexed formats may need to be coupled to local secretome or downstream molecular measurements when both the interaction and its consequence must be resolved. Rapid drug-response testing instead favors controlled perturbation and repeated live-cell measurement, with endpoint molecular profiling added when the observed response needs to be connected to the state of the same cell. A hybrid workflow becomes useful when the biological question requires several platform functions to be integrated online or multiple forms of information continuity that cannot be maintained reliably by one platform alone; otherwise, additional integration may increase sample loss and calibration burden without improving the biological answer. Figure 7 summarizes this selection process, distinguishing single-function from hybrid platforms at the first decision point and direct readouts from source-linked analysis at the second (Figure 7).

**Table 2:** Biological Question-Guided Selection of Microfluidic Strategies and Hybrid Workflows for Single-Cell Tumor Heterogeneity Analysis.

Biological Question	Heterogeneity to Be Resolved	Microfluidic Principle	Suitable Device Design/Methodology	Resulting Measurement/Application	Selection Caveat/Hybrid Trigger	Refs
Which single cells generate divergent clones or longitudinal phenotypes?	Clonal and temporal heterogeneity	Spatial indexing and retention of cell identity	Microwell or microchamber arrays	Clonal tracking, organoid formation, longitudinal imaging	Limited retention of diffusible products; add local capture/flow control when needed	[32,59]
Which cells produce distinct secretory or metabolic outputs?	Secretory and metabolic heterogeneity	Compartmentalization and cell-to-output attribution	Droplet microfluidics	Single-cell EV, protein, metabolite, or enzymatic-output analysis	Limited reagent exchange and recovery; add indexed recovery/downstream profiling for cross-layer linkage	[66,75]
How do individual cells respond dynamically to defined perturbations?	Dynamic therapy-response and state-transition heterogeneity	Programmable temporal control of stimulation and reagent exchange	Valve-assisted chips	Timed stimulation, sequential treatment, and time-resolved response analysis	Greater complexity and lower throughput; add molecular profiling for response–state linkage	[63,64]
Which rare cells or vesicle populations can be recovered from limited clinical samples?	Rare-cell and circulating-population heterogeneity	Selective enrichment and recovery from low-input samples	Capture chips	CTC or EV enrichment followed by downstream phenotypic or molecular analysis	Marker dependence and capture bias; capture alone does not define cell state	[46,76]
Can physical or electrical properties distinguish heterogeneous cell states rapidly?	Biophysical heterogeneity	Label-free interrogation within a defined sensing region	Electrical or impedance chips	Rapid single-cell classification and biophysical phenotyping	Limited molecular specificity; add orthogonal molecular/protein validation for state assignment	[69,70]
How do molecular, physical, secretory, and functional states align within the same biological unit?	Multimodal and cross-layer heterogeneity	Preservation of identity across sequential analytical steps	Integrated multimodal chips	Linked functional–molecular profiling and drug-response analysis	Added modules increase sample loss and calibration burden; use only when cross-layer linkage is essential	[77,78]

## 4. Major Readout Modalities in Microfluidic Single-Cell Analysis of Tumor Heterogeneity

Microfluidic single-cell analysis is valuable in tumor heterogeneity research because it preserves distinct biological signals at single-cell resolution. Different readout modalities interrogate partially overlapping tumor-cell populations. The same microfluidic architecture can therefore support different levels of biological interpretation depending on the readout coupled to it, while the same readout may yield different information when cell identity, spatial context, or perturbation history has been preserved to different degrees upstream. The analytical task is to establish which biological layer each signal represents and how it relates to disease behavior. The analytical challenge is therefore to determine which layer of heterogeneity is being measured and how that layer contributes to tumor architecture, progression, and adaptation [79]. For this reason, a biologically oriented classification of microfluidic single-cell readouts is often more informative than one based only on device architecture (Table 3). The following sections focus on four major modalities: genomic and transcriptomic profiling, EV analysis, metabolic and functional phenotyping, and protein and secretome analysis [80]. These modalities are methodologically distinct but biologically complementary. Genomic and transcriptomic profiling resolves clonal structure and regulatory state, EV analysis captures source-resolved communication, metabolic and functional phenotyping measures cellular performance, and protein and secretome analysis reflects functional output and interaction-dependent signaling. Together, these readouts provide a more complete picture of tumor heterogeneity than any single information layer alone [81]. A critical distinction across these readout modalities is the boundary between on-chip information preservation and off-chip analytical amplification. In sequencing-linked or mass-spectrometry-linked workflows, the main contribution of microfluidics is often not the final molecular detection itself, but the preservation of cell identity, compartmental origin, perturbation history, or low-input sample integrity before downstream analysis. By contrast, optical, electrical, barcode-based, and plasmonic platforms can convert cell-derived signals more directly into on-chip or chip-coupled readouts. Clarifying this boundary prevents the analytical power of downstream technologies from being attributed entirely to the microfluidic device, and it helps define which part of the workflow should be optimized when reproducibility, sensitivity, or clinical translation remains limited. Because each readout captures only one layer of tumor heterogeneity, interpretation should focus on what the signal can and cannot support. A transcriptomic state should not be automatically interpreted as functional resistance, increased EV release should not be assumed to represent regulated communication without excluding stress or viability effects, and label-free electrical or optical classification should not be treated as mechanistic evidence without orthogonal validation. For this reason, readout modalities should be evaluated by their attribution level, interpretation boundary, common sources of overinterpretation, and minimum validation requirements.

### 4.1. Genomic and Transcriptomic Profiling at Single-Cell Resolution

Single-cell genomic and transcriptomic profiling is foundational to the study of tumor heterogeneity because it resolves two related but distinct questions: how malignant cells are organized into subclones and how those cells differ in regulatory state. In microfluidic systems, this readout is used to profile mutations, copy number alterations, and gene expression programs at single-cell resolution, making it possible to recover rare subclones and state variation that would be obscured in pooled measurements. Its particular value is the ability to interpret clonal structure and state diversity within the same analytical framework [82,89]. At the genomic level, microfluidic enrichment is most valuable when rare tumor cells must be recovered and profiled with minimal sample loss. Oyama et al. developed a targeted sequencing workflow for single cells captured on a polymeric microfluidic CTC device. Using the Universal CTC-chip and a direct sequencing strategy that bypasses whole-genome amplification, they carried out mutation analysis on single lung cancer cells and then applied the workflow to patient blood samples in a liquid-biopsy context [76]. In this setting, microfluidics enabled direct mutation-oriented profiling of rare recovered cells despite intrinsically limited input material.

**Figure 8:**
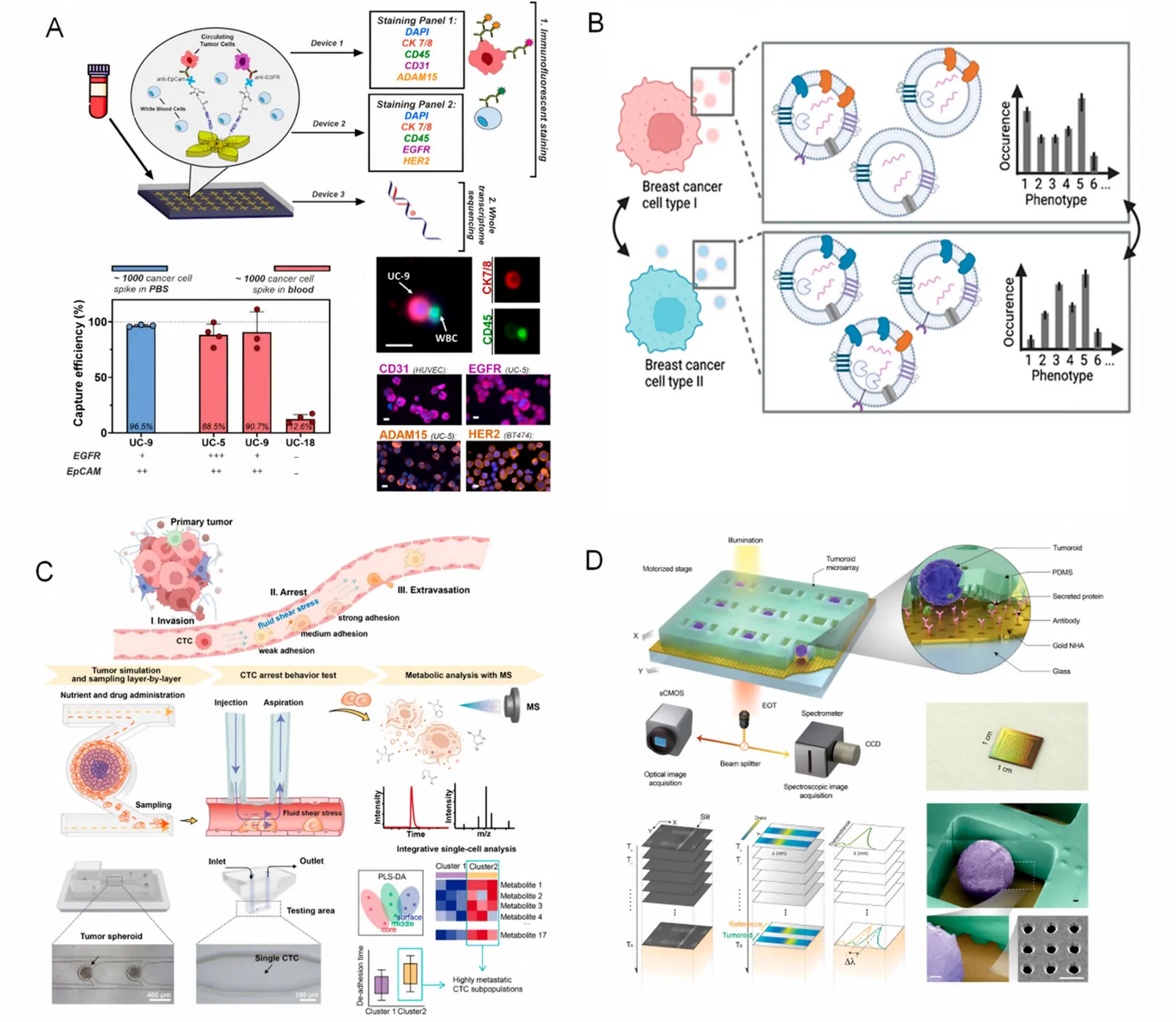
Representative readout modalities used in microfluidic platforms for tumor-related single-cell analysis. Here, +, ++, and +++ indicate increasing relative expression levels of EGFR and EpCAM, whereas − indicates no detectable expression. (**A**) Immunofluorescence-based protein readout for circulating tumor cell (CTC) capture and phenotypic characterization, enabling multiplexed detection of tumor-associated markers at the single-cell level. Reproduced in part with permission from Ref. [45]. Copyright 2024, Elsevier Inc. (**B**) Heterogeneous exosomal receptor-expression phenotypes and their cell-type-specific frequency distributions in breast cancer. Reproduced in part with permission from Ref. [53]. Copyright 2025, Elsevier B.V. (**C**) Mass spectrometry (MS)-based metabolic readout integrated with a microfluidic model of CTC invasion, arrest, and extravasation for single-cell metabolomic profiling and identification of metastatic subpopulations. Reproduced in part from Ref. [102]. Copyright 2025, the author(s). Published by Wiley-VCH GmbH. Licensed under CC BY 4.0. (**D**) Spectroscopic imaging-based secretome protein readout in a tumoroid microarray, enabling spatially resolved detection of tumor-associated secreted proteins and microenvironmental heterogeneity. Reproduced in part from Ref. [54]. Copyright 2024, the author(s). Published by Wiley-VCH GmbH. Licensed under CC BY 4.0.

Genomic information alone, however, does not explain why cells within the same disease setting differ in phenotype or clinical behavior. Niu et al. addressed this question using a graphene oxide microfluidic chip functionalized with EpCAM and EGFR antibodies to capture circulating tumor cells from bladder cancer patients. After capture from 1 mL of patient blood, the workflow incorporated whole-transcriptome sequencing alongside protein and mRNA analysis (Figure 8A). The transcriptomic data revealed differences between patients with and without tumor burden at the time of blood draw, and CTCs from patients with advanced metastatic disease showed increased expression of metastasis-related and chemotherapy-resistant genes [45]. This provides a state-level view of how rare circulating tumor cells vary molecularly across disease conditions. Recent sequencing-linked workflows have further sought to retain contextual information that is normally lost during tissue dissociation. King et al. used single-cell transfection-enabled cell hashing (scTECH-seq) to encode the spatial origin of cells within engineered three-dimensional tumor spheroids before droplet-based single-cell RNA sequencing. The retained spatial barcodes allowed transcriptional responses to drug treatment to be related back to defined regions of the spheroid, revealing spatially dependent heterogeneity in drug response. This approach illustrates a broader progression from identifying genomic or transcriptional differences among isolated tumor cells toward preserving contextual information alongside single-cell molecular profiles [90].

Taken together, these studies show why genomic and transcriptomic profiling is a foundational readout in microfluidic single-cell tumor research. Genomic measurements define the structural composition of rare malignant populations, whereas transcriptomic measurements reveal how those populations diversify in state. The strength of this modality is its ability to place these two levels within the same analytical framework, although genomic and transcriptomic information alone does not fully capture downstream functional behavior. Nevertheless, genomic and transcriptomic readouts should not be interpreted as complete representations of tumor behavior. Genomic analysis can define clonal structure, but it may be affected by allele dropout, amplification bias, limited genome coverage, or low-input sequencing noise. Transcriptomic analysis provides a sensitive view of cell state, but it can be influenced by tissue dissociation, capture-marker selection, cell viability, and processing time. In many microfluidic workflows, the chip preserves rare cells or cell identity before sequencing, whereas the final molecular amplification and data generation occur off chip. The biological interpretation therefore depends on whether microfluidic recovery, sequencing quality, and downstream functional validation remain aligned. Transcriptomic variation should therefore be interpreted as a state-level hypothesis rather than direct evidence of functional resistance or metastatic behavior unless it is paired with perturbation, protein-level, or functional validation.

### 4.2. Extracellular Vesicles as a Distinct Communication Layer

EVs provide a distinct communication-layer readout in tumor heterogeneity research. Unlike soluble cytokines or metabolites, EVs are particulate carriers that can preserve combinations of membrane proteins, cytosolic proteins, nucleic acids, and lipids derived from their parental cells. This makes EV profiling particularly useful for resolving communication-associated heterogeneity, but it also makes the readout highly sensitive to compartment design, EV retention, capture chemistry, and marker selection [91,92]. At single-cell resolution, the central question is not only how many EVs are released, but whether EV phenotype, release dynamics, and cargo-associated signatures remain attributable to a defined cellular source. Microfluidic platforms address this challenge by confining individual cells, retaining locally secreted EVs, and enabling capture close to the source cell [93,94,95,96].

Nikoloff et al. demonstrated this principle with a microfluidic platform for profiling EVs from single breast cancer cells. The system isolated individual cells, immobilized secreted EVs while preserving source attribution, and used four-color total internal reflection fluorescence microscopy to enumerate EVs and define biochemical fingerprints based on membrane and cytosolic protein markers. Here, cell-of-origin assignment was preserved directly by the device architecture: individual cells were physically isolated, their EVs were captured within the corresponding local compartment, and the vesicles were analyzed in situ rather than pooled and reassigned after off-chip processing. EVs released by single cells were sufficient to distinguish cell-type-specific patterns across breast cancer and nonmalignant comparator cells, although interpretation still depended on the selected marker panel and capture strategy [46,97]. Once source attribution is secured, EV profiling can also distinguish heterogeneous cellular states rather than merely catalog EV output. Wang et al. developed a SERS-based microfluidic platform for single-cell analysis of breast epithelial and breast cancer cells through exosome-associated signatures. Their system profiled EV receptor expression at single-cell resolution, enabled discrimination among cell types, and, when combined with machine learning, assessed subtype-associated EV signatures after drug treatment [53] (Figure 8B). At the EV-sensing level, Gil et al. combined SERS with graphene field-effect transistor sensing to obtain complementary optical and electrical signatures from breast- and colorectal-cancer-derived exosomes using a tag-free graphene platform [98]. Although this approach did not preserve single-cell source attribution, it illustrates how SERS can be coupled with an orthogonal electrical readout to broaden EV phenotyping. The single-cell SERS platform described above therefore extended EV analysis beyond descriptive enumeration by resolving treatment-associated and subtype-dependent differences between cells, although interpretation still depended on capture chemistry, spectral features, and model generalizability. Dynamic perturbation adds a temporal dimension to EV profiling, because changes in EV release can reveal treatment-associated communication states that static measurements may miss. Pei et al. developed a chip for longitudinal quantification of EV release from individual cells during drug treatment. EV secretion correlated closely with drug sensitivity and varied strongly with dose: low-dose treatment increased secretion, whereas high-dose treatment reduced both viability and EV output. The study also identified a slowly fluctuating secretion pattern in stem-like cells and provided evidence that EVs from drug-resistant cells could facilitate resistance transfer in co-culture [52]. These results highlight a treatment-responsive communication layer that pooled measurements are ill-suited to capture, although secretion changes under drug exposure must be interpreted cautiously because they may reflect both regulated EV output and viability-related effects.

EV analysis therefore contributes to tumor heterogeneity research in a manner distinct from other secretory readouts. It links EV features to cells of origin, resolves heterogeneous cellular states through EV-associated signatures, and captures how EV-mediated communication changes during perturbation. Its specific strength is the preservation of a source-resolved communication layer that can be interpreted alongside genomic, transcriptomic, proteomic, and functional measurements [99,100]. Despite these advantages, source-attributed EV analysis remains technically and biologically challenging. EV retention, capture efficiency, marker selection, and compartment leakage can all influence the apparent EV phenotype assigned to a single cell. Changes in EV release under drug treatment may reflect regulated intercellular communication, cellular stress, reduced viability, or altered EV biogenesis, and these possibilities are difficult to distinguish from EV measurements alone. Single-cell EV readouts should therefore be interpreted together with parental-cell phenotype, viability, perturbation history, and, where possible, orthogonal molecular or functional measurements. This caution is particularly important when EV profiles are used to infer treatment response, resistance transfer, or communication between heterogeneous tumor subpopulations. For source-attributed single-cell EV assays in particular, compartment leakage or cross-contamination, capture-marker specificity, and preservation of cell-of-origin assignment require explicit validation, because failure at any of these steps can create an apparent single-cell EV phenotype that does not reliably represent the producing cell.

### 4.3. Metabolic and Functional Phenotypes of Heterogeneous Tumor Cells

Metabolic and functional phenotyping is especially informative when molecular identity alone is insufficient to explain tumor heterogeneity. Genomic, transcriptomic, and proteomic measurements define key aspects of cellular identity and regulatory state, but they do not fully explain how cells behave under physical constraint, during migration, or in response to selective pressure. Microfluidic platforms are particularly useful in this context because they confine, perturb, and monitor individual cells in controlled microscale environments, making differences in metabolic state and behavior experimentally accessible [83,84].

One important contribution of this modality is that it resolves metabolic heterogeneity directly at single-cell resolution. Xing et al. developed a workflow that combined droplet-generated hydrogel microspheres for single-cell encapsulation, a microchamber culture chip, and downstream mass spectrometry. In this workflow, the hydrogel microsphere served as the physical carrier of single-cell identity across the processing steps: individual cell-containing microspheres were isolated in separate microchambers before downstream MS analysis, rather than pooling the cultured material before measurement. Single-cell metabolic heterogeneity was then resolved from separately processed microspheres, including 23 individual A549-cell measurements. Using this system, they measured metabolite accumulation from individual tumor cells, detected heterogeneity within the same tumor cell type, and distinguished metabolic patterns among A549, HepG2, and HCT116 cells. In this context, metabolism could be measured at single-cell resolution within a three-dimensional culture setting. Throughput was sufficient to capture cell-to-cell variation, although the resulting metabolic state still depends on culture conditions and measurement timing [101]. This type of physical indexing is important because downstream MS analysis does not itself preserve cellular provenance; the single-cell assignment remains credible only when the microsphere, compartment, spatial index, or molecular barcode can be traced through the transfer and detection steps. Metabolic readouts gain interpretive value when they are linked to measurable tumor-cell behavior, such as migration, invasion, or metastatic competence. Hou et al. addressed this by combining single-cell behavioral analysis with metabolic profiling in spheroid-derived circulating tumor cells. Their integrated workflow used a multifunctional microfluidic chip to simulate aspects of the metastatic process and coupled this with single-cell mass spectrometry [102]. The resulting design enabled examination of behavioral and metabolic heterogeneity within the same analytical framework to determine whether metabolic differences tracked with metastatic behavior (Figure 8C).

Microfluidic functional phenotyping can also distinguish tumor-cell subpopulations directly by their behavior, even before those behaviors are interpreted molecularly. Ding et al. developed an automated microfluidic platform that sorted cancer cells according to migration phenotype and validated the system with MDA-MB-231 and MCF-7 cells. Sorting was completed within 6.5 h, and subsequent SMART-seq2 analysis of the resulting high- and low-migration subpopulations identified differentially expressed genes and migration-related pathways [103]. Here, behavior itself served as a practical basis for subpopulation separation, enabling subsequent examination of underlying molecular programs, although migration phenotype remains operationally defined by the assay conditions used for sorting. A more explicit functional readout emerges when invasion-related capacity is quantified through cell deformation and channel traversal rather than inferred from secondary measurements. Bhattacharya et al. developed a high-throughput microfabricated microchannel assay in which individual cells actively deformed and crossed constrictions narrower than the nuclear diameter. Using MCF-7 cells and the more metastatic LM2-4 line, they defined an invasion index, showed a marked difference between the two cell types, and adapted the platform to a 96-well format for drug testing [104]. Accordingly, the platform provided a direct quantitative readout of invasion-related behavior under controlled physical constraints, although deformation-based traversal should be regarded as a functional proxy rather than a full representation of in vivo invasion. Recent work has extended this mechanical phenotype from measurement to direct cell enrichment. Liu et al. developed an ultrahigh-throughput microfluidic platform that sorts cancer cells according to deformability and subsequently characterized the mechanically separated subpopulations by RNA sequencing. This moves deformability beyond a phenotype recorded after isolation: the mechanical property itself becomes the basis for recovering distinct tumor-cell fractions for downstream biological analysis. The study therefore provides a direct example of how a biophysical difference can be used to resolve metastasis-associated heterogeneity rather than serving only as a surrogate measurement of invasive behavior [105].

Overall, metabolic and functional phenotyping extends microfluidic single-cell analysis from molecular description to measurable cellular performance. These readouts identify which cells differ in metabolic output, migration, deformation, invasion-associated behavior, or drug response capacity, and they can reveal heterogeneity that is not obvious from molecular profiles alone. Their main limitation is that functional phenotypes are operationally defined by the assay conditions used to measure them. Nutrient composition, matrix stiffness, confinement geometry, shear exposure, culture duration, measurement timing, and rapid ex vivo phenotypic drift in primary biopsy material can substantially influence the observed behavior. Therefore, metabolic and functional readouts should be interpreted as controlled proxies for tumor-cell performance rather than direct replicas of in vivo tumor behavior, unless they are linked to orthogonal molecular, imaging, or clinical evidence.

### 4.4. Protein and Secretome Analysis

Single-cell protein and secretome analysis is especially informative when tumor heterogeneity must be understood at the level of functional output, beyond molecular identity alone. Genomic and transcriptomic measurements are indispensable for defining clonal architecture and regulatory state, but they do not directly reveal how individual tumor cells signal, respond, or interact with neighboring cells. At single-cell resolution, differences in protein abundance and secretory activity provide a more immediate view of the functional output of heterogeneous tumor cells within the tumor microenvironment [86,106,107].

Microfluidic platforms are particularly useful for this purpose because they confine individual cells in small volumes and retain locally secreted molecules near their source, thereby preserving source attribution for secreted and interaction-dependent signals that would otherwise be diluted in pooled assays. As a result, secretory heterogeneity, inducible responses, and local protein states can remain assigned to individual cells rather than averaged across populations [87,88]. Wang et al. developed a high-throughput platform for profiling multiple secreted biomarkers from living single tumor cells. Their system combined single-cell culture chambers with an antibody barcode chip and machine-learning analysis, enabling quantitative secretome profiling from thousands of individual cells and supporting accurate tumor-cell classification. More importantly for tumor heterogeneity research, secretion-based grouping resolved subgroup-specific output patterns that would be difficult to recover from pooled measurements [77]. In this setting, secretome profiling exposed functional differences between cells through what they secreted. Protein-level analysis can also be extended from isolated tumor cells to defined cell–cell interactions, where the measured proteomic state reflects both intrinsic cellular programs and contact-dependent signaling [108]. Xu et al. developed a microfluidic single-cell-pair proteomics platform that integrated precise cell pairing, on-chip co-culture, real-time microscopic monitoring, pair retrieval, and mass-spectrometry-based analysis. Using single NK cells paired with single K562 cells, they identified more than 1000 protein groups in a single cell pair and resolved heterogeneous NK-response subclusters with associated protein signatures [78]. This extended protein readout from tumor-cell-intrinsic output to interaction-dependent proteomic states. Longitudinal secretome monitoring adds dynamic context by linking protein secretion to growth, movement, hypoxia, and drug exposure in the same tumoroid or compartment. Liu et al. addressed this with a nanoplasmonic single-tumoroid microarray that integrated an ultrasensitive biosensor with a PDMS microwell array for label-free, real-time analysis of individual colorectal tumoroids (Figure 8D). The platform enabled simultaneous observation of VEGF-A secretion, growth, and movement under normoxia, hypoxia, and drug treatment, and it was applied to both cell-line-derived and patient-derived tumoroids [54]. Here, protein secretion emerged as a dynamic phenotype shaped by microenvironmental and therapeutic context. Protein and secretome readouts provide a direct view of functional output, but they also introduce interpretation challenges. Antibody-barcode and capture-based platforms are usually limited to predefined protein panels, and measured signals can be affected by antibody specificity, surface adsorption, secretion kinetics, and local cell density. Secretory bursts may reflect stable functional programs, transient stress responses, paracrine feedback, or changes in viability. In interacting cell-pair or tumoroid systems, attribution can become more complex because the measured protein landscape may reflect both tumor-cell-intrinsic activity and microenvironment-dependent signaling. These limitations do not reduce the value of protein and secretome profiling, but they indicate that such readouts are most informative when combined with cell identity, viability, spatial context, and perturbation history.

For EV and soluble-secretome measurements, we consider several controls to be the minimum needed before a change in release can be interpreted as regulated signaling. Cell viability and evidence of cell death or lysis should be assessed under the same perturbation and sampling conditions as the secretory readout, and secretion should be normalized to the number of viable cells or another explicitly stated cellular denominator. Untreated or vehicle-matched controls are needed to establish the perturbation-dependent baseline, while no-cell/background controls and, for compartmentalized assays, leakage or cross-contamination controls are needed to exclude signals introduced by the medium, device, or transfer process. The specificity and calibration of the capture or detection interface should also be reported, together with evidence that the measured output remains attributable to the intended cell or compartment. Importantly, an increase in EV or protein release should not by itself be taken as evidence of regulated communication if it coincides with loss of viability or membrane integrity. Where regulated signaling is a central conclusion, its temporal relationship to the perturbation and, where possible, an orthogonal EV, protein, or functional readout provide stronger support for that interpretation.

Taken together, these readout modalities should be viewed as complementary rather than hierarchical. No single modality simultaneously establishes cellular identity, functional output, and clinical relevance. Multimodal integration is useful only when measurements remain attributable to the same cell, clone, compartment, perturbation condition, or multicellular interaction. Multimodal integration is therefore most informative when it links these layers within a coherent biological framework, rather than simply increasing the number of measured variables. However, true single-cell multimodal integration must address the physical conflict between destructive molecular profiling, such as cell lysis for sequencing, and non-destructive longitudinal monitoring, such as live-cell secretion, migration, or drug-response analysis [109]. The success of an integrated workflow therefore depends on how these analytical steps are ordered, so that cellular viability and functional information are preserved for as long as possible before end-point molecular profiling. A sensible way to arrange these workflows is to order measurements according to how irreversible each one is. Any readout that requires viable cells and repeated access to the same biological unit should be performed before fixation, lysis, or other terminal steps. Baseline morphology, viability, migration, deformation, label-free biophysical signals, and time-resolved secretion can therefore be recorded while cell identity and experimental history remain traceable, followed by controlled perturbation and longitudinal response monitoring where needed. The same cell or indexed clonal unit can then be recovered or transferred without losing its identifier, before destructive molecular profiling, such as single-cell transcriptomic or genomic analysis, is performed as the final endpoint. This ordering becomes especially important when the aim is to connect a molecular state to a preceding functional behavior rather than simply measure both in the same sample. If the identity of the same cell cannot be maintained through the destructive step, this limitation should be stated explicitly rather than implying a direct functional–molecular link from parallel or population-level measurements. This distinction is central to applications that require interpretation of dissemination, tumor–microenvironment interaction, therapy response, and drug resistance [110,111]. The value of a platform for these applications therefore depends on whether its architecture preserves the information required by the question and whether the selected readout can support the intended biological interpretation. This combined design–readout constraint provides the basis for evaluating the biomedical applications discussed in the following section.

## 5. Biomedical Applications in Tumor Heterogeneity Research

The transition from analytical capability to biomedical application requires the device architecture and readout modality to be matched to the biological information needed for a specific question. The biomedical value of microfluidic single-cell analysis should not be assessed only by whether a platform detects more markers, processes fewer cells, or generates more single-cell data. Its translational significance depends on whether the measured heterogeneity can inform a defined biological or clinical question. In circulating tumor cell analysis, the relevant issue is whether rare circulating states can be linked to dissemination, recurrence, or treatment adaptation. In TME modeling, microfluidics is most useful when selected stromal, immune, matrix, or vascular-like interactions can be made experimentally controllable. In therapy-response testing, the critical requirement is to preserve resistant or adaptive fractions within a clinically meaningful time window. This decision-oriented perspective is essential for distinguishing technically impressive demonstrations from platforms with translational relevance. However, translational relevance also requires validation against clinically meaningful endpoints, because a platform that resolves heterogeneity ex vivo does not automatically demonstrate diagnostic, prognostic, or therapeutic utility. The level of evidence required for clinical interpretation depends on the intended use of the platform. For exploratory tumor biology, it may be sufficient to show that a microfluidic readout resolves reproducible cell-state or functional heterogeneity under controlled conditions. For biomarker discovery, the same readout should be linked to independent molecular, imaging, or pathological evidence. For prognostic interpretation, chip-derived states must correlate with clinically meaningful outcomes such as recurrence, metastasis, progression-free survival, or overall survival in adequately powered cohorts. For treatment selection, ex vivo response metrics must be prospectively compared with patient response to therapy within clinically actionable time windows. This distinction is essential because a platform can be biologically informative without yet being clinically actionable. Moving from these levels of evidence to clinical action requires the validation design to follow the intended claim. For biomarker discovery, candidate chip-derived signatures should be defined in a discovery cohort and then tested in an independent cohort with the marker panel, model, and decision threshold fixed before validation. Prognostic studies should use prespecified clinical endpoints and be powered at the patient level rather than by the number of cells profiled; the required sample size should reflect the expected number of outcome events, effect size, and precision of the primary estimate, with recurrence, metastasis, progression-free survival, or overall survival defined in advance [112]. Treatment-selection claims require a higher level of evidence. Ex vivo response should be prospectively compared with the treatment actually received, and claims that a microfluidic readout identifies differential treatment benefit are best tested in a randomized or biomarker-stratified design that can distinguish a predictive effect from a purely prognostic association [113]. Short-term endpoints may include objective or pathological response, whereas progression-free survival, recurrence, and overall survival provide stronger evidence of longer-term clinical benefit. Sample-size planning should also account for assay failure, insufficient specimen yield, and the prevalence of the rare cell state being evaluated. Importantly, profiling thousands of cells from a small number of patients does not provide patient-level statistical power.

### 5.1. Clonal Evolution and Dissemination in Rare Circulating Tumor Cell Populations

**Figure 9:**
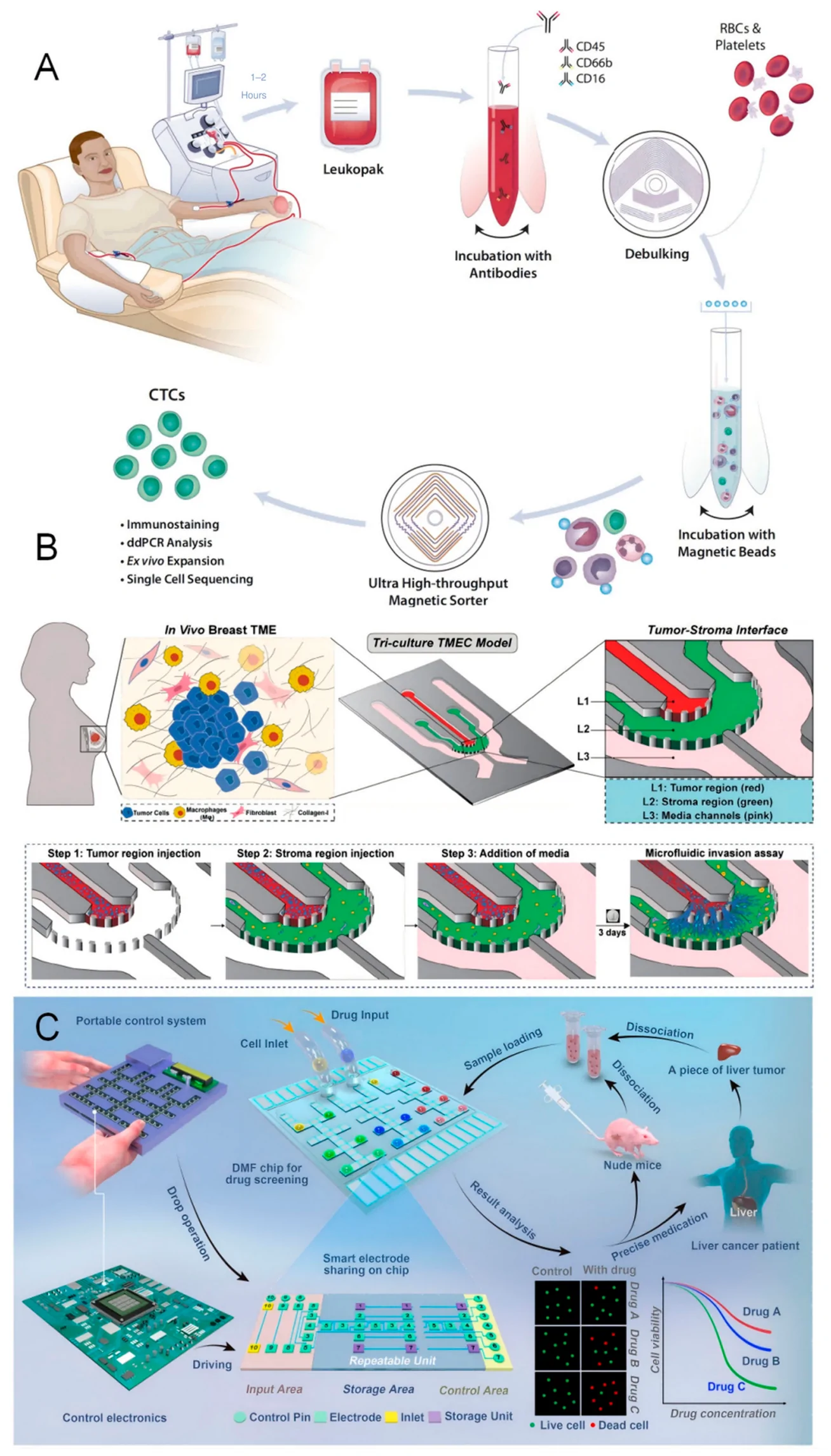
Representative biomedical applications of microfluidic technologies in tumor heterogeneity research. (**A**) High-throughput enrichment and isolation of circulating tumor cells (CTCs) from blood-derived samples through antibody-assisted magnetic sorting, enabling downstream immunostaining, ddPCR analysis, ex vivo expansion, and single-cell sequencing. Reproduced in part from Ref. [118]. Copyright 2025, the author(s). Licensed under CC BY 4.0. (**B**) Microfluidic tri-culture TME model for reconstructing the tumor–stroma interface and investigating tumor cell invasion, stromal interactions, and heterogeneity-related metastatic behaviors. Reproduced in part from Ref. [127]. Copyright 2025, the author(s). Published by Wiley-VCH GmbH. Licensed under CC BY 4.0. (**C**) Digital microfluidic platform for patient-derived tumor cell drug screening, integrating sample dissociation, programmable droplet manipulation, on-chip drug treatment, and viability-based response analysis to support precision oncology. Reproduced in part from Ref. [134]. Copyright 2024, the author(s). Licensed under CC BY 4.0.

In circulating tumor cell research, microfluidic single-cell analysis is shifting the field from enumeration to state-resolved discrimination. The central question is no longer only how many CTCs are present, but which rare circulating states carry information about clonal evolution, dissemination, recurrence, or treatment adaptation. This requires rare circulating cells to be recovered at sufficient scale and quality to resolve clonal structure, state variation, and differences in clinical relevance. Total circulating tumor cell burden is often a poor surrogate for biological importance because the fractions most relevant to clonal evolution, recurrence, or dissemination are frequently rare, plastic, and unevenly represented [114,115,116,117]. Mishra et al. showed how expanded rare-cell recovery can broaden the scope of CTC analysis. Their high-throughput microfluidic enrichment workflow was applied to patient-derived leukapheresis products instead of standard small-volume peripheral blood, allowing processing of a mean blood-equivalent volume of 5.83 L and recovery of substantially increased CTC numbers [118]. In spike-in validation of the leukapheresis-capable system, the reported capture efficiency was 86.1 ± 0.6%, providing a practical reference for the recovery achievable under optimized conditions. This value should not, however, be interpreted as the true recovery of patient-derived CTCs, because the number and phenotypic composition of CTCs entering a clinical sample are not known. The substantially increased sampling volume expanded what could be resolved analytically (Figure 9A). Paired single-cell DNA and RNA sequencing identified subclonal aneuploidy patterns and distinct signaling programs within circulating tumor cells, and in prostate cancer the analysis uncovered a rare neuroendocrine-signature population lacking conventional epithelial markers [119,120]. This large-scale recovery made rare circulating subclones and cell states accessible to joint genomic and transcriptomic analysis [121]. Zheng et al. shifted the focus from broad circulating composition to rare fractions defined by biological state. Using an integrated immunomagnetic-microfluidic platform, they investigated circulating tumor-initiating cells (CTICs) in hepatocellular carcinoma (HCC) and resolved four stem-related CTIC subsets. The platform also enabled these subsets to be tracked during treatment, showing that their relative composition differed across primary, recurrent, and transarterial chemoembolization (TACE)-resistant HCC [47]. By resolving biologically distinct CTIC subsets, the platform enabled tracking of rare circulating fractions associated with recurrence and treatment adaptation, rather than reducing circulating heterogeneity to abundance alone. Dissemination-oriented applications require a stricter question: which circulating fractions predict metastatic risk more effectively than total CTC burden. Zhang et al. used a single-cell quantitative mass spectrometry platform to profile colorectal-cancer-derived CTCs and developed a molecular typing system based on metabolic fingerprints. A metabolically defined CTC subgroup was more informative for metastasis prediction than total CTC count, and the framework was further examined in both test and independent prospective cohorts. Here, single-cell microfluidic analysis linked rare circulating states to dissemination-associated risk more directly than bulk CTC enumeration [122].

Microfluidic CTC analysis is most valuable when rare-cell recovery is converted into clinically interpretable state information. Its contribution is not simply the detection of more circulating cells, but the ability to link rare circulating fractions to clonal structure, regulatory state, dissemination-associated risk, and treatment adaptation. Nevertheless, CTC profiling is inherently shaped by selection bias. Cells detected in blood represent the fraction that has entered circulation, survived hemodynamic stress, avoided immune clearance, and remained compatible with the enrichment strategy. They therefore cannot be assumed to fully represent the heterogeneity of the primary tumor or metastatic lesions. Sampling volume, marker selection, cell viability, enrichment bias, and the difference between prognostic association and clinical actionability must be addressed before CTC-state profiling can be treated as a robust decision-making tool [123,124].

### 5.2. Microfluidic Dissection of Tumor–Microenvironment Interactions

In tumor microenvironment (TME) research, the goal of microfluidic modeling is not to reproduce the entire tumor ecosystem, but to make selected interaction variables experimentally controllable. Tumor heterogeneity is continually generated and reshaped through interactions with stromal cells, immune cells, extracellular matrix, and soluble signals [125]. Microfluidic systems are especially useful in this setting because they can preserve or reconstruct these interactions under controlled conditions while maintaining greater experimental observability than conventional culture. The central question is therefore how heterogeneity emerges through interaction context, in addition to tumor-cell-intrinsic variation [126].

For multicellular reconstruction, Ravi et al. developed an organotypic three-dimensional breast TME chip incorporating triple-negative breast cancer cells, patient-derived cancer-associated fibroblasts (CAFs), and macrophages, and analyzed the resulting system by single-cell RNA sequencing. Functional assays showed that fibroblasts and macrophages synergistically increased invasion and altered morphology, while transcriptomic and pathway analyses implicated KYNU and the kynurenine pathway as a candidate immune-evasion axis (Figure 9B). This platform enabled mechanistic inference about multicellular stromal–immune–tumor crosstalk in a reconstructed microenvironment [127]. This type of model is most useful when the question concerns how a reconstructed stromal or immune context reshapes tumor-cell state at the population and pathway level. When the goal is to resolve heterogeneity generated by individual immune-tumor encounters, a more granular pairing strategy becomes necessary. Flores et al. used a microfluidic integrated fluidic circuit to capture cancer cells and immune cells, co-incubate defined doublets, perform time-lapse imaging, and recover gene expression profiles from the same cell pairs [128]. Relative to the Ravi study, the focus shifted from multicellular architecture to single-encounter heterogeneity. This study showed that transcriptional divergence can reflect both broad microenvironmental composition and the dynamics of specific immune–tumor contacts. Interaction heterogeneity can also unfold over time, particularly when immune recruitment and cooperative killing shape the final tumor response. Ronteix et al. extended the temporal dimension further through the MIOCS platform, which enabled high-resolution tracking of antigen-specific T-cell activity against many individual tumor spheroids together with probabilistic modeling [129]. The study showed that early T-cell recruitment accelerated later accumulation and that cooperative behavior among T cells facilitated tumor killing. Here, heterogeneity emerged as a dynamically organized collective process rather than a purely pairwise interaction outcome.

Microfluidic TME models are most informative when they isolate a defined interaction variable rather than attempt to reproduce the full tumor ecosystem. They can resolve heterogeneity arising from stromal support, immune contact, matrix context, vascular-like interfaces, and collective dynamics, but their interpretation remains constrained by model reduction. Shear stress in microchannels, artificial matrix stiffness, simplified vascular geometry, oxygen and nutrient gradients, and the absence of native interstitial pressure can all diverge substantially from in vivo tumor tissue. Missing systemic immune, endocrine, lymphatic, and pharmacokinetic inputs further limit direct clinical extrapolation. Therefore, TME-chip findings should be interpreted as controlled mechanistic models of selected interactions, rather than complete replicas of tumor ecology in vivo.

### 5.3. Functional Prediction of Therapy Response and Resistance Evolution

In therapy-response studies, the central translational question is whether ex vivo microfluidic assays can preserve clinically relevant resistant fractions within a clinically actionable time window. Therapeutic response is one of the clearest translational applications of microfluidic single-cell analysis because clinically meaningful resistance often resides in rare or adaptable fractions that conventional assays fail to preserve [130]. The central issue is whether an ex vivo platform can preserve the heterogeneity that determines response, adaptation, and relapse, instead of reducing drug response to an average effect [131,132,133].

At a basic translational level, Zhai et al. developed a digital microfluidic system for drug screening on primary tumor cells using as few as 100 cells per drug condition (Figure 9C). This study showed that response testing remained feasible even when available material was far below the requirements of conventional plate-based screening, a major practical barrier in precision oncology [134]. Fang et al. extended this logic from low-input feasibility to the preservation of response heterogeneity during testing. They developed a microarray platform for high-throughput three-dimensional culture of breast-cancer patient-derived organoids and used it to study adriamycin resistance. Coupled with fluorescence monitoring, the platform enabled on-chip drug-response assessment without consuming large numbers of tumor cells [135]. The key advance was preserving inter-organoid response heterogeneity during functional testing. Resistance, however, is not always tumor-intrinsic. Low-input feasibility and preservation of response heterogeneity are necessary but not sufficient for clinical translation. A therapy-response platform must also define what its endpoint means, whether it measures viability, apoptosis, growth delay, secretion change, molecular adaptation, or clonal outgrowth. Without a clearly defined endpoint, apparent drug sensitivity may be difficult to compare across platforms or align with clinical response. Ro et al. addressed this by developing the ODSEI chip, an open 3D microarray platform capable of arraying more than 1000 tumor spheroids in a vascularized setting and enabling single-spheroid-level analysis of drug resistance. In their breast-cancer model, endothelial interaction contributed to acquired tamoxifen resistance. This expanded resistance analysis from tumor-cell-intrinsic heterogeneity to microenvironment-induced adaptive resistance [136]. However, vascularized or endothelial-interaction models still simplify systemic pharmacokinetics, immune regulation, and stromal diversity, all of which can influence treatment response in vivo. Recent platforms have also begun to bring these workflows closer to patient-linked prediction by combining viable CTC capture with in situ molecular and phenotypic analysis on the same cells. The NICHE device is an example of this direction, designed to enable both gene-expression and phenotypic analysis of single living CTCs in situ. This illustrates how heterogeneity-aware ex vivo profiling may support patient-oriented response assessment, although clinical utility will require prospective validation against treatment outcomes [137,138].

Microfluidic single-cell systems contribute to therapy-response research at several distinct levels, including low-input feasibility, preservation of resistant heterogeneity during testing, analysis of context-dependent resistance, and movement toward patient-linked predictive workflows. Their translational value, however, depends on more than technical feasibility. Ex vivo drug-response assays cannot fully reproduce pharmacokinetics, hepatic metabolism, plasma protein binding, systemic immune clearance, or host-level toxicity, all of which can determine clinical response in patients. Future platforms must therefore demonstrate that their response metrics remain stable within clinically actionable time windows, reflect clinically relevant mechanisms of resistance, and correlate with treatment outcomes in prospective patient cohorts. Without this validation, microfluidic drug-response assays should be regarded as powerful functional profiling tools rather than established clinical decision systems [139,140,141]. A recurring translational risk is that technically sophisticated platforms may not be the most clinically useful platforms. Systems with high-dimensional readouts, complex microenvironmental reconstruction, or elaborate multistep integration can reveal important biology, but they may be difficult to standardize, slow to operate, costly to manufacture, or incompatible with limited patient material. Conversely, a simpler platform with fewer readouts may have greater translational value if it produces a robust, interpretable, and clinically timed endpoint. Therefore, translational potential should be judged by the match between the platform, the clinical question, sample availability, assay duration, endpoint clarity, and validation strategy, rather than by technical complexity alone. The same distinction applies to validation for clinical use. Demonstrating stable recovery, signal reproducibility, or agreement with an established analytical method shows that a platform can measure its intended endpoint, but does not by itself establish that the endpoint is clinically informative. Once an assay is proposed for diagnosis, prognosis, or treatment selection, the relevant question becomes whether the chip-derived measurement retains its association with the intended clinical outcome in independent and, where appropriate, prospectively defined patient cohorts. Regulatory requirements follow the same logic: the pathway depends on the intended use, device classification, and the type of clinical claim being made, with routes such as 510(k), De Novo, or premarket approval applying under different circumstances in the United States. These considerations also bring seemingly secondary engineering choices back into platform design. Material selection, manual transfer steps, fluidic automation, assay turnaround time, and manufacturing complexity affect both cost and the likelihood that an assay can be reproduced outside the laboratory in which it was developed, and should therefore be considered before analytical performance has been fully optimized.

## 6. Current Bottlenecks and Future Directions for Translational Microfluidic Single-Cell Oncology

High-resolution measurement of heterogeneous tumor cells is no longer the main barrier for microfluidic single-cell analysis in tumor heterogeneity research. This capability has been demonstrated across platforms that recover rare cells, preserve local signals, reconstruct selected microenvironmental interactions, and link single-cell states to molecular or functional readouts. The more difficult barrier is translational credibility: whether these measurements remain stable across sample handling, reproducible across platforms, biologically representative of clinically relevant tumor states, and validated against endpoints that matter for patient care [142]. Accordingly, the current bottlenecks arise at the intersection of sample integrity, analytical design, biological representation, and clinical validation [143,144].

### 6.1. Bottlenecks in Reproducibility, Biological Representation, and Clinical Interpretation

A first set of constraints concerns reproducibility and comparability. Preanalytical variation remains a major problem because cell recovery, viability, and molecular integrity are strongly affected by sampling method, storage interval, preservation condition, enrichment strategy, and downstream processing. These variables should not be treated as peripheral technical noise. In low-input single-cell assays, they can reshape the measured cell population, distort transcriptional or secretory states, and alter functional responses before the chip-based measurement even begins. Even when platform performance is strong under controlled conditions, such variation can determine what is ultimately measured. This makes comparison across studies difficult and complicates interpretation of whether observed differences reflect tumor heterogeneity or preanalytical handling [145,146]. This means that preanalytical handling should be considered part of assay validation rather than a fixed step preceding the microfluidic workflow. For measurements intended to resolve rare or transient states, recovery and viability alone are not sufficient; it is also necessary to determine whether the state being interpreted remains stable between sampling and measurement. Time-controlled processing experiments or paired measurements before and after enrichment could help distinguish biological variation from state changes introduced by the workflow itself. These challenges are further shaped by the architecture of current platforms, many of which still impose a trade-off between scale and depth. Devices optimized for rare-cell enrichment or throughput do not necessarily preserve the same degree of functional context as platforms designed for dynamic co-culture, longitudinal imaging, or multistep perturbation. A platform may therefore isolate rare populations efficiently yet still provide limited information about how those cells behave, interact, or adapt over time [75]. This trade-off becomes a translational problem when platforms are compared without regard to their intended use. A system optimized for CTC enumeration should not be judged by the same criteria as a system designed for therapy-response prediction or microenvironmental mechanism discovery [147]. Device fabrication, signal-processing pipelines, and reporting metrics remain insufficiently harmonized across the field, which slows cross-platform comparison and cross-institutional validation. At the translational level, this becomes even more consequential because many elegant systems are still supported mainly by proof-of-concept cohorts. The link between chip-derived measurements and clinical endpoints remains underdeveloped, especially when the intended use is prediction, stratification, or treatment selection rather than mechanistic exploration [148]. These observations also argue against optimizing all platforms toward the same technical endpoint. What needs to be demonstrated depends on the claim the platform is intended to support. For CTC enumeration, reliable recovery and classification may be sufficient, whereas a platform used to interpret treatment response must additionally show that the measured state is preserved during processing and remains associated with subsequent functional or clinical behavior. Once the latter claim is made, further improvement in throughput or analytical complexity is less informative than testing whether the same relationship can be reproduced in independent samples or cohorts.

Technical reproducibility alone, however, will not solve the central problem if the biological scope of the assay remains too narrow. A reproducible assay can still be biologically incomplete. Many current devices capture only one layer of heterogeneity at a time, although clinically consequential behavior often emerges from interactions among multiple layers. Tumor progression and treatment resistance depend on how copy number variation, transcriptional state, secretory phenotype, metabolic state, mechanical behavior, immune interaction, and drug response interact within individual cells and across multicellular ecosystems. In addition, model systems frequently compress the tumor environment into simplified representations of vasculature, immune composition, extracellular matrix mechanics, nutrient supply, oxygen tension, and evolutionary pressure. This model compression is experimentally useful because it makes selected variables controllable, but it also limits direct extrapolation to patient tumors. A platform may therefore measure heterogeneity accurately at the level it was designed for, yet still fail to capture the biological context in which that heterogeneity becomes clinically consequential. The solution is not necessarily to reproduce more of the tumor environment in every experiment. Simplified systems remain valuable when they are used to isolate a defined interaction or causal variable. The stronger test is whether the relationship identified under controlled conditions remains detectable when the same assay is applied to patient-derived cells, organoids, tumor fragments, or more complex multicellular models. In this sense, biological representation is better treated as a staged validation problem than as a requirement for maximal model complexity from the outset.

### 6.2. Benchmarking and Reporting Standards

A major limitation of the field is the lack of consistent benchmarking across microfluidic single-cell platforms [149]. Many studies report platform performance using different sample types, cell numbers, efficiency definitions, and downstream endpoints. As a result, it is often difficult to determine whether differences between studies reflect device performance, biological variation, or preanalytical handling. This limitation is particularly important for tumor heterogeneity research, where rare populations, transient states, and low-abundance secreted signals are highly sensitive to sampling and processing conditions. The field therefore needs a Minimum Information standard for microfluidic single-cell tumor analysis, analogous in spirit to reporting frameworks used in other high-dimensional biological technologies. Such a standard should not prescribe a single device format, but should define the minimal experimental, biological, and clinical-context information required for a study to be interpretable, reproducible, and comparable across laboratories [150,151,152]. Without this level of disclosure, apparent differences in rare-cell recovery, secretion profiles, drug response, or molecular state may reflect platform-specific artifacts rather than genuine tumor heterogeneity.

Material- and surface-related bias should therefore be reported as a measurable part of platform performance rather than only as a fabrication detail. At minimum, studies should specify the substrate and coating chemistry, surface-treatment procedure, relevant storage or aging conditions, and the surface-to-volume exposure experienced by the sample. Bias can be quantified using defined reference cells or analytes by comparing input with post-device recovery, nonspecific adsorption or background signal, and, where relevant, changes in viability or cellular state after surface contact [153,154]. For capture or antifouling interfaces, target recovery and nonspecific retention should be reported separately, while EV, protein, and secretome assays should include controls for surface-associated analyte loss. These measurements should be repeated across independent devices and, where possible, different fabrication or coating batches, with the corresponding variability reported explicitly [155]. Contact time, flow conditions, and sample matrix should also be documented so that differences between laboratories can be separated more readily from material- or interface-dependent effects.

Standardized reporting would also make platform comparisons more meaningful, but benchmarking should not imply that all platforms should be ranked on a single universal scale. A droplet platform designed for high-throughput compartmentalization should not be directly compared with a valve-assisted platform designed for temporal control unless the biological question and required information are defined. Similarly, a capture chip optimized for CTC enrichment should be evaluated differently from a multimodal workflow designed to link biophysical phenotype with transcriptomic state. Benchmarking should therefore be organized around intended use, including rare-cell recovery, source-attributed secretion analysis, dynamic perturbation, multimodal linkage, or clinical response prediction [156]. The relevant standard is not whether one platform is universally superior, but whether it preserves the information required for a specific tumor-heterogeneity problem.

### 6.3. Future Directions: From Descriptive Profiling to Decision-Oriented Microfluidic Systems

Future progress will depend on building stronger continuity between measurement, biological interpretation, and clinical use. Three developments are particularly important. The first is multimodal integration that links genomic, transcriptomic, proteomic, metabolic, and functional phenotypes within the same cell, clone, EV-producing unit, or multicellular interaction [157]. The second is longitudinal sampling, especially in liquid-biopsy and therapy-monitoring settings, where changing clonal structures and therapy-selected cell states must be followed through treatment rather than inferred from isolated time points [158]. The most informative next-generation platforms will likely combine rare-cell enrichment, dynamic perturbation, and multilevel readout within workflows that remain practical under clinical constraints [159,160,161]. The third is the closer integration of microfluidic systems with patient-derived materials and disease-relevant microenvironments. Single-cell-derived organoids, circulating tumor cells, intact microtumor fragments, and multicellular TME chips each point toward a future in which patient-specific heterogeneity is measured in functional terms and within clinically relevant time windows [162,163]. These capabilities open several possibilities for questions that remain difficult to address with conventional endpoint assays. For example, microfluidic workflows can monitor live-cell behavior and secretion during controlled drug exposure, potentially revealing transient therapy-adaptive states before they develop into stable resistance, while allowing the same cells to be recovered for downstream molecular analysis. They are also well suited to source-resolved studies of rare secretory events, in which treatment-induced EVs, cytokines, or other signals remain traceable to the individual cell or defined cellular interaction from which they originated. In longitudinal liquid-biopsy settings, serial sampling could move beyond changes in cell abundance to determine whether rare circulating populations also change state as treatment proceeds. What links these applications is that the relevant biological information is distributed across time, source, and cellular identity and therefore cannot be fully reconstructed from a single endpoint measurement. In this context, the value of microfluidics lies not simply in assay miniaturization, but in preserving these relationships throughout the measurement process. Computational and machine-learning approaches will become more useful as microfluidic workflows generate increasingly heterogeneous data, but their value depends on how those data were linked experimentally. Measurements obtained from the same cell can be integrated directly only when cell identity is preserved across modalities. When molecular, imaging, secretory, or functional measurements come from different cells or sampling points, combining them does not recreate a true single-cell multimodal profile. Computational deconvolution or population-level integration may still be informative in these cases, but the level of attribution should remain explicit. Dataset size requires similar caution. Thousands of cells obtained from a small number of patients provide substantial cellular information but limited patient-level independence, and random cell-wise splitting may therefore overestimate model generalizability. Capture bias, preanalytical variation, batch effects, and platform-specific signatures can also become predictive features when training and validation data share the same technical dependencies. Model performance should therefore be evaluated together with cohort structure, batch structure, cell-of-origin attribution, and independent validation rather than by classification accuracy alone [164,165]. At this stage, simply adding more readout layers is unlikely to resolve the main limitations of microfluidic single-cell analysis. Each additional operation can also introduce cell loss, state drift, or a break in the linkage between measurements, which is particularly problematic when the population of interest is rare or transient. A more useful question for future platform design may therefore be how much biological linkage can be retained as analytical complexity increases. In this regard, sequential workflows may be more practical than fully simultaneous multimodal systems for many applications. Rare cells could first be recovered with minimal perturbation, followed by live-cell functional or temporal measurements and, only after the relevant phenotype has been observed, endpoint molecular profiling. Such an order would allow molecular states to be interpreted against an experimentally observed behavior rather than inferred from molecular data alone.

Over the longer term, the field should move toward systems that can identify which heterogeneous subpopulations are relevant for monitoring, intervention, and therapeutic stratification [166]. The critical transition is from descriptive profiling to decision-oriented microfluidic systems in which heterogeneity informs a defined biological or clinical action. This transition will require platforms that minimize the loss or distortion of rare cells, transient states, and cell-derived products during processing, while also disclosing the capture surfaces, hydrogel matrices, droplet interfaces, sensing elements, and computational pipelines that can introduce measurement bias. It will also require workflows that connect molecular, functional, and temporal readouts to the same cell, clone, EV-producing unit, or multicellular interaction whenever possible. Most importantly, chip-derived metrics must be anchored to defined clinical questions, including dissemination risk, therapy selection, minimal residual disease, resistance monitoring, and patient outcome prediction. The next stage of the field should therefore not be defined by higher throughput or more markers alone, but by the ability to preserve clinically meaningful heterogeneity and convert it into reproducible, interpretable, and decision-relevant information.

## 7. Conclusions

Microfluidic single-cell analysis is most informative when it preserves the relationships that give each measurement biological meaning, including cell identity, signal source, local context, perturbation history, and response. Different microfluidic architectures retain these relationships in different ways: microwell and microchamber systems favor spatial identity and longitudinal observation, droplet platforms provide compartment-based source attribution, and valve-assisted or integrated systems offer greater control over perturbation and multistep analysis. Their value ultimately depends on how well these capabilities are matched to the readout and to the biological question being addressed. Genomic, transcriptomic, extracellular-vesicle, secretomic, metabolic, and functional measurements therefore provide complementary rather than interchangeable views of tumor heterogeneity. For questions involving treatment response or disease evolution, longitudinal sampling and sequential workflows that preserve viable-cell phenotypes before endpoint molecular profiling may be particularly useful for maintaining the link between observed behavior and molecular state. In this context, a decision-oriented system is not simply one that measures more cells or more markers, but one designed around the information needed for a defined biological or clinical decision and validated accordingly. Further progress will depend on whether microfluidic platforms can preserve this information reliably across processing, measurement, and clinical application, allowing heterogeneous tumor states to be interpreted rather than merely detected.

## Figures and Tables

**Table 1:** Selected microfluidic engineering elements that influence information preservation in single-cell heterogeneity analysis.

Design Element	Representative Implementations	Main Microfluidic Function	Single-Cell Information Preserved	Best-Suited Application	Main Bias or Limitation	Refs
Substrate materials	PDMS, glass, thermoplastics	Determine optical access, gas transport, bonding and manufacturability	Live-cell dynamics and longitudinal phenotypes	Live-cell culture, imaging, valve systems and tumor-on-chip models	Molecular adsorption, material-dependent gas transport and scale-up constraints	[37,38]
Flow and separation architectures	Hydrodynamic focusing, inertial or Dean-flow separation, geometry-based channels	Enrich, sort or position cells by physical properties	Rare-cell recovery, population composition and biophysical phenotype	Label-free CTC enrichment and upstream sample preparation	Physical-property bias, shear stress and clogging	[39]
Spatial indexing and confinement	Microwells and microchambers	Retain cells at defined positions	Cell identity, clonal origin and longitudinal response	Clonal tracking, organoid formation and repeated imaging	Loss of diffusible products and limited reagent exchange or recovery	[40,41]
Active fluidic control	Pneumatic valves, programmable reagent routing and pressure-driven transfer	Control reagent delivery and sequential operations	Temporal history and perturbation–response linkage	Sequential stimulation, dynamic drug-response analysis and multistep workflows	Instrumental complexity, dead volume and lower throughput	[42]
Antifouling surfaces	PEG, zwitterionic coatings and BSA blocking	Reduce nonspecific adsorption and background	Low-abundance protein, EV and secretome signals	Clinical samples, immunoassays and secretion analysis	Limited coating durability, batch variability and incomplete fouling resistance	[43,44]
Recognition interfaces	Antibodies, aptamers and peptides	Selectively capture cells, EVs or proteins	Rare-cell identity, EV phenotype and marker-defined subpopulations	CTC capture, EV enrichment and protein profiling	Marker bias, loss of marker-low populations and affinity-dependent recovery	[45,46,47]
Hydrogel or ECM interfaces	Collagen, Matrigel, GelMA and PEG hydrogels	Provide mechanical and biochemical cues	Invasion, matrix response, drug adaptation and TME-associated phenotypes	Three-dimensional culture, organoids, tumor-on-chip models and drug screening	Batch variability, diffusion limits and matrix-induced phenotypic changes	[32,48,49]
Droplet interfaces	Oil–water, all-aqueous and hydrogel droplets	Isolate cells and retain diffusible outputs	Compartment identity and source-attributed secretome, EVs and metabolites	Secretion, EV, sequencing and organoid assays	Doublets, leakage, cellular stress, limited exchange and difficult recovery	[50,51,52]
Sensing interfaces	Electrodes, SERS or plasmonic surfaces, barcodes and optical elements	Convert cellular signals into measurable outputs	Electrical phenotype, protein abundance, EV signatures and secretory output	Impedance, SERS, barcode and optical detection	Calibration drift, restricted specificity and chip-to-chip variability	[53,54]

**Table 3:** Readout Modalities, Interpretation Risks, and Validation Requirements in Microfluidic Single-Cell Tumor Analysis.

Readout Modality	Heterogeneity Layer Resolved	Main Microfluidic Contribution	Interpretation Boundary	Common Overinterpretation	Minimum Validation Needed	Refs
Genomic and transcriptomic profiling	Clonal structure and regulatory state	Rare-cell recovery, compartment identity, low-input processing	Sequencing bias, capture bias, and limited functional inference	Treating transcriptional state as direct functional behavior	Viability/state preservation controls, orthogonal protein or functional validation	[45,76,82]
EV analysis	Source-resolved communication	Cell confinement, EV retention, local capture	Marker dependence, leakage, viability effects, and stress-induced release	Treating increased EV release as regulated communication without excluding stress or death	Viability/death or lysis controls; viable-cell normalization; leakage/cross-contamination controls; capture specificity; source-attribution validation	[42,75,76]
Metabolic and functional phenotyping	Cellular performance and adaptive behavior	Controlled microenvironment, perturbation, live-cell monitoring	Assay-defined proxies and condition-dependent behavior	Treating migration, deformation, or metabolic accumulation as direct in vivo behavior	Matrix/geometry controls, orthogonal molecular readouts, clinically relevant perturbation conditions	[83,84]
Protein and secretome analysis	Functional output and interaction-dependent signaling	Local protein capture, small-volume confinement, paired-cell analysis	Predefined panels, adsorption, secretion kinetics, viability/stress effects, and attribution complexity	Interpreting increased protein release as regulated signaling without excluding stress, cell death, or changes in viable-cell number	Viability/death or lysis controls; viable-cell normalization; no-cell/background controls; capture specificity and calibration; source-attribution validation	[54,85,86,87,88]

## Data Availability

No new data were created or analyzed in this study. Data sharing is not applicable to this article.

## Abbreviations

Abbreviation	Full Term
CTC/CTCs	Circulating tumor cell(s)
EV/EVs	Extracellular vesicle(s)
ECM	Extracellular matrix
PDMS	Polydimethylsiloxane
PEG	Poly(ethylene glycol)
BSA	Bovine serum albumin
GelMA	Gelatin methacryloyl
TME	Tumor microenvironment
SERS	Surface-enhanced Raman scattering
DMSO	Dimethyl sulfoxide
IC-ECIS	Integrated circuit-electric cell–substrate impedance sensing
PI	Propidium iodide
EpCAM	Epithelial cell adhesion molecule
EGFR	Epidermal growth factor receptor
MS	Mass spectrometry
NK cells	Natural killer cells
VEGF-A	Vascular endothelial growth factor A
CTIC/CTICs	Circulating tumor-initiating cell(s)
HCC	Hepatocellular carcinoma
TACE	Transarterial chemoembolization
CAFs	Cancer-associated fibroblasts
ddPCR	Droplet digital polymerase chain reaction
